# Selective binding of retrotransposons by ZFP352 facilitates the timely dissolution of totipotency network

**DOI:** 10.1038/s41467-023-39344-1

**Published:** 2023-06-20

**Authors:** Zhengyi Li, Haiyan Xu, Jiaqun Li, Xiao Xu, Junjiao Wang, Danya Wu, Jiateng Zhang, Juan Liu, Ziwei Xue, Guankai Zhan, Bobby Cheng Peow Tan, Di Chen, Yun-Shen Chan, Huck Hui Ng, Wanlu Liu, Chih-Hung Hsu, Dan Zhang, Yang Shen, Hongqing Liang

**Affiliations:** 1 Division of Human Reproduction and Developmental Genetics, Women’s Hospital, and Institute of Genetics, Zhejiang University School of Medicine, Hangzhou, 310006 China; 2 Key Laboratory of Reproductive Genetics (Ministry of Education) and Department of Reproductive Endocrinology, Women’s Hospital, School of Medicine, Zhejiang University, Hangzhou, 310006 China; 3 Zhejiang Provincial Clinical Research Center for Child Health, Women’s Hospital, School of Medicine, Zhejiang University, Hangzhou, 310006 China; 4 Department of Orthopedic Surgery of the Second Affiliated Hospital of Zhejiang University School of Medicine, Zhejiang University, Hangzhou, Zhejiang 310003 China; 5 Zhejiang University-University of Edinburgh Institute (ZJU-UoE Institute), Zhejiang University School of Medicine, International Campus, Zhejiang University, 718 East Haizhou Rd., Haining, 314400 China; 6 Women’s Hospital, Institute of Genetics, and Department of Environmental Medicine, Zhejiang University School of Medicine, Hangzhou, 310006 China; 7 Laboratory of Precision Disease Therapeutics, Genome Institute of Singapore, Agency for Science, Technology and Research (A*STAR), 60 Biopolis Street, 138672 Singapore, Singapore; 8 Guangzhou Laboratory, Guangzhou International Bio Island, Guangzhou, 510005 Guangdong Province China; 9 Department of Biological Sciences, National University of Singapore, 14 Science Drive 4, 117597 Singapore, Singapore; 10 School of Biological Sciences, Nanyang Technological University, 60 Nanyang Drive, Singapore, 639798 Singapore; 11 Present Address: Vision Medicals Co., Ltd, G10 BLDG, Huaxin Park, 31 Kefeng Ave, Gaungzhou, 510000 China

**Keywords:** Totipotent stem cells, Gene regulation, DNA transposable elements, Transcriptional regulatory elements, Embryonic induction

## Abstract

Acquisition of new stem cell fates relies on the dissolution of the prior regulatory network sustaining the existing cell fates. Currently, extensive insights have been revealed for the totipotency regulatory network around the zygotic genome activation (ZGA) period. However, how the dissolution of the totipotency network is triggered to ensure the timely embryonic development following ZGA is largely unknown. In this study, we identify the unexpected role of a highly expressed 2-cell (2C) embryo specific transcription factor, ZFP352, in facilitating the dissolution of the totipotency network. We find that ZFP352 has selective binding towards two different retrotransposon sub-families. ZFP352 coordinates with DUX to bind the 2C specific MT2_Mm sub-family. On the other hand, without DUX, ZFP352 switches affinity to bind extensively onto SINE_B1/Alu sub-family. This leads to the activation of later developmental programs like ubiquitination pathways, to facilitate the dissolution of the 2C state. Correspondingly, depleting ZFP352 in mouse embryos delays the 2C to morula transition process. Thus, through a shift of binding from MT2_Mm to SINE_B1/Alu, ZFP352 can trigger spontaneous dissolution of the totipotency network. Our study highlights the importance of different retrotransposons sub-families in facilitating the timely and programmed cell fates transition during early embryogenesis.

## Abstract (web summary)

During zygotic genome activation the embryo must re-wire the regulatory network that sustains totipotency earlier during development. Here they identify ZFP352 as an essential factor that targets retrotransposon families to facilitate dissolution of the totipotency network and enable ZGA.

## Introduction

Stem cell fates transition consists of two inter-dependent processes: demolishing of the existing cell fates and emergence of the new cell fates. While the activation of regulatory pathways and genes dictating new cell fate plays pivoting roles, the dissolution of the prior cell state is of paramount importance. Precise triggering and co-ordination between the two events are the foundation for accurate and efficient cell fate transition. As revealed from previous studies, the dissolution of existing stem cell fates is not a passive dilution process, but rather requires active molecular machineries. For instance, the dissolution of pluripotency state precedes lineage differentiation^1^ and is under the precise coordination of transcription, protein degradation, and cell cycle machineries^1–3^. Similarly, in the early embryos after ZGA, cells gradually exit from the totipotent state^4^ to make the first lineage choice diverging into extra-embryonic fates or inner cell mass^5^. How the dissolution of the totipotency network is triggered and whether the exit from totipotent state involves specific regulatory machineries remain largely unknown.

The identification of mouse totipotent-like cells marked by the MERVL reporter activation has provided a surrogate in vitro totipotency model for the 2-Cell (2C) stage embryos^6^, and it has greatly extended our understanding towards totipotency regulation^7–15^. Various mechanisms have been reported to reprogram pluripotent mouse embryonic stem cells (mESCs) into totipotent-like state^16–26^. The current understanding of totipotency transcription is converged to a few 2C specific transcription factors like DUX, RARG, and ZSCAN4 family^8,9,27–30^. Upon activation, these transcription factors, which are at the central node of the 2C transcription network, can re-enforce one another’s expression and also rewire the expression of other 2C genes^8,27,28,31,32^. Subsequently, with the activation of 4-Cell (4C) and later developmental genes, the totipotency transcription network sustaining the 2C embryo is destabilized. Yet, how the shifting in transcription states from 2C to later stage embryos is initiated molecularly, and whether transcription independent programs are involved in the totipotency dissolution process currently remain understudied.

In the mammalian genome, retrotransposons consist of significant proportion in the non-coding genome^33–36^, and they have been shown to elicit functions under various physical and pathological conditions^15,37–42^. During early embryogenesis, different classes and families of retrotransposons are activated with high developmental-stage specificities^39,43^. While chromatin decompaction in early embryogenesis serves as a permissive condition to potentiate retrotransposon activation^36^, transcription factors may be the pivoting driver for the precise and temporal activation of various retrotransposon sub-families. MT2_Mm/MERVL sub-family is specifically activated by totipotency transcription factors^8,14,28^ and has been used as a 2C stage specific marker^7^. MT2_Mm/MERVL has also been functionally implicated in the 2C transcription network^44^. Yet how other sub-families of retrotransposons are specifically activated in early mouse embryos, and whether they specialize in different roles in shaping the cell state-specific function and potency during early embryogenesis have not been revealed extensively.

In this work, we showed that one of the top expressed 2C transcription factors, ZFP352 has unexpected roles in the 2C transcription network. ZFP352 has binding affinity towards two different retrotransposons. We find that ZFP352 coordinates with DUX to bind on MT2_Mm sub-family in 2C state. On the other hand, when DUX expression is low, ZFP352 alone exhibits extensive binding onto SINE_B1/Alu sub-family, activating later developmental genes nearby SINE_B1/Alu to facilitate the dissolution of 2C state. Our study reveals the mechanism of how the dynamic wiring of a transcription factor, like ZFP352, through different retrotransposon sub-families can facilitate the timely and programmed cell fates transition out of the totipotent state.

## Results

### ZFP352 promotes the destabilization of 2-Cell like state

**Fig. 1:**
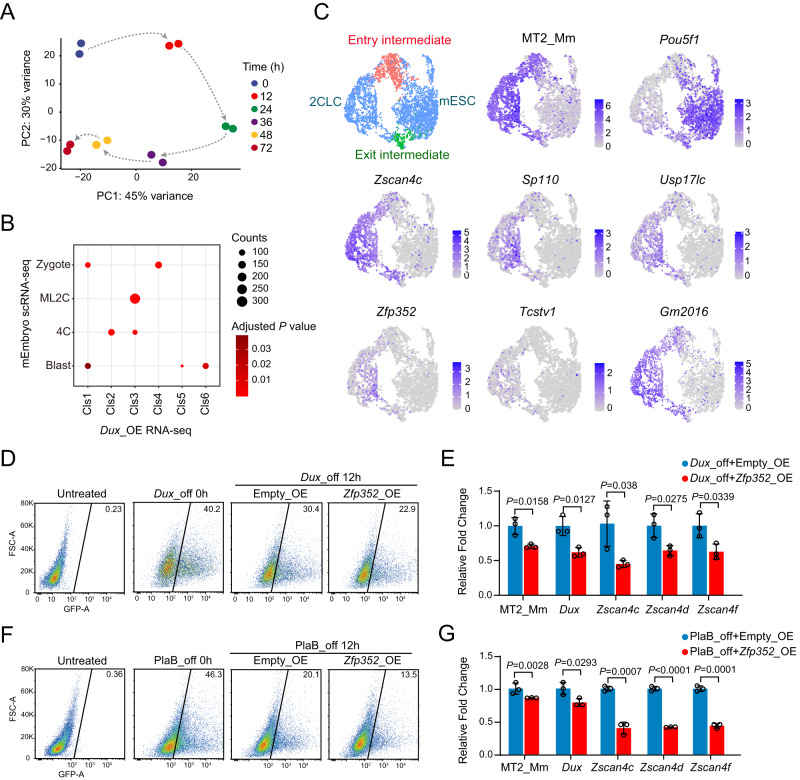
ZFP352 is involved in the destabilization of 2CL state. **A** Principal component analysis (PCA) revealing the dynamic changes in the transcriptome over different times upon *Dux* induction. RNA-seq was performed to analyze the transcriptome changes in mESCs with *Dux* overexpression for 0, 12, 24, 36, 48, and 72 h. **B** Enrichment analysis showing the enrichment of different DUX-induced DEGs clusters (Extended Data Fig. 1C) with embryo stage specific gene clusters. The size of the dot represents number of overlapped genes in the clusters, and color code indicates Adjusted *P* value for the significance of overlap. Only the overlap with Adjusted *P* value < 0.05 were shown (one-sided hypergeometric test, multiple test by Benjamini & Hochberg method, ML_2C: mid_late 2C, Blast: blastocyst). **C** Uniform manifold approximation and project (UMAP) for single-cell transcriptome of mESCs integrated from two datasets: (1) dataset for 2CLC entry induced by *Dux* overexpression (GSE121459), (2) dataset for exit from DUX-induced 2CLC (GSE133234). The feature plots of MT2_Mm, *Pou5f1*, *Zscan4c*, *Usp17lc*, *Zfp352*, *Pou5f1*, *Sp110*, *Tcstv1*, *Gm2016* were shown in the respective UMAPs. Entry and exit intermediate population were highlighted as red and green in the UMAP. **D**, **E** *Zfp352* was over-expressed when cells were released from DUX-induced 2CL state by withdrawal of doxycycline to switch off exogenous *Dux* expression. The effect of *Zfp352* overexpression during the exit from 2CL state was measured by the percentage of MERVL positive 2CLC population (MERVL::dsGFP mESC line) from FACS (**D**). RT-qPCR was also performed to measure the relative expression changes of key endogenous 2C markers, MT2_Mm, *Dux*, *Zscan4c*, *Zscan4d, Zscan4f* upon *Zfp352* overexpression (**E**). **F**, **G** *Zfp352* was over-expressed in cells released from PlaB induced TBLC state by withdrawal of PlaB. The effect of *Zfp352* overexpression during the exit from TBLC state was measured by the percentage of MERVL positive 2CLC population by FACS (**F**), and the relative expression changes of key 2C markers were measured by RT-qPCR (**G**). RT-qPCR data in **E**, **G** were presented as mean ± SD, *n* = 3 biologically independent samples, two-sided unpaired t-test.

The mouse embryonic stem cells fluctuate into and out of the 2-Cell like (2CL) state. The spontaneous and prominent exit from natural occurring or induced 2CL state promoted us to explore the underlying causes^28,45^. A few pathways have recently been implicated in mediating the exit from 2CL state, including the nonsense-mediated decay of DUX mRNA, activation of RNA splicing, or rRNA biogenesis^16–21,45^. However, how these totipotency-repressive pathways can be initiated while in the totipotent state remains unknown. Hence, we explored the potential causes using the 2CL state induced by exogenous DUX expression. DUX-induced 2CL state was previously documented to be highly dynamic, and it exhibited spontaneous exit phenotype which partially mimics the developmental transition after 2C stage^45^. Upon overexpression of exogenous DUX (Supplementary Fig. 1A), MT2_Mm, the long terminal repeat (LTR) of 2C specific MERVL, underwent dynamic activation and dampening within 72 h (Supplementary Fig. 1B). The temporal expression changes of MT2_Mm also corresponded with the dynamic changes in the whole transcriptome (Fig. 1A). We analyzed the differentially expressed genes (DEGs) induced by exogenous DUX, and clustered them based on the temporal expression pattern upon DUX induction (Supplementary Fig. 1C, Supplementary Data 1 and Supplementary Data 2). Down-regulated DEGs upon DUX induction (DUX_cls1) overlapped with zygotic and blastocyst genes in mouse embryos^46^ (Fig. 1B).

Region-associated DEGs analysis (RAD)^47^ was used to assess the enrichment of DUX_cls1 DEGs around DUX ChIP peaks in mESCs^8^ (Supplementary Fig. 1D), and the lack of significant enrichment suggests the down-regulation of these genes may not be directly controlled by DUX binding. Up-regulated DEGs within 12–24 h after DUX induction (DUX_cls2 and DUX_cls3) (Supplementary Fig. 1C) significantly overlapped with mid_late 2C and 4C genes (Fig. 1B). These DEGs were highly enriched nearby DUX ChIP peaks (Supplementary Fig. 1D), suggesting DUX directly bound and activated them. However, DUX_cls2&3 DEGs were silenced subsequently after 36 h (Supplementary Fig. 1C), while DUX_cls4,5,6 DEGs became gradually up-regulated (Supplementary Fig. 1C). In contrast with DUX_cls2&3, DUX_cls4,5,6 DEGs neither enriched with 2C specific genes (Fig. 1B), nor located nearby DUX ChIP peaks (Supplementary Fig. 1D). They were instead enriched with blastocyst and zygotic genes (Fig. 1B), reflecting the involvement of DUX binding independent transcription in activating the later developmental programs. These temporal expression profiles thus imply that the transition out of the 2CL state to pluripotent ESC states may not be directly triggered by DUX itself.

In order to identify potential regulators of the later developmental programs, we analyzed the single-cell RNA-seq profiling data during the entry and exit from DUX-induced 2CL state^15,45^, to look for dynamic expression patterns of DUX up-regulated 2C genes (Supplementary Fig. 1E). Interestingly, one of the top five enriched mid_late 2C genes^46^ (Supplementary Fig. 1F), ZFP352, was only up-regulated in a sub-population of MERVL positive cells, close to the “exit intermediate” state (Fig. 1C). The “exit intermediate” state represents cells in the process of transiting from 2CL state into ESC state^45^. ZFP352 is a transposon derived zinc finger transcription factor highly specific and abundantly expressed in 2C stage^7,8,48–50^; yet the function of ZFP352 in the 2C transcription network was unknown.

This prompted us to explore the impact of ZFP352 in the DUX-induced 2CL totipotency network. To investigate whether ZFP352 interacts and coordinates with DUX, ZFP352 was over-expressed in DUX-induced 2C-like cells (2CLCs) (Supplementary Fig. 2A, B). Surprisingly, except for MT2_Mm expression, other 2C-specific transcription factors, like endogenous *Dux* and *Zscan4* family were all down-regulated (Supplementary Fig. 2C), while exogenous DUX expression did not change (Supplementary Fig. 2D). This implies a potential antagonizing effect of ZFP352 on DUX-induced 2CL state. Conversely, when knocking down *Zfp352* in DUX-induced 2CLCs (Supplementary Fig. 2E), up-regulation of MT2_Mm and 2C transcription factors was evident (Supplementary Fig. 2F), while in this case syn*Dux* RNA level also increased (Supplementary Fig. 2D). Given that syn*Dux* expression from DOX promoter should be stable in the inducible cell line, it is not clear whether syn*Dux* RNA stability was under regulation in this case, and whether the up-regulation of 2C markers was a direct effect by *Zfp352* knocking down or indirectly through the reduction of syn*Dux* RNA.

Thus, to further validate whether ZFP352 has a role in the totipotency exit process, the exit from DUX-induced 2CL state or exit from Pladienolide (PlaB) induced totipotent blastomere-like cell (TBLC) state was tested^8,45^. Exogenous DUX induction was withdrawn to allow the rapid exit from 2CL state (Supplementary Fig. 2G), and with *Zfp352* overexpression (Supplementary Fig. 2H), a faster decrease of the MERVL positive population (Fig. 1D), as well as hastened down-regulation of 2C markers, like MT2_Mm, endogenous *Dux* and *Zscan4* family (Fig. 1E), were observed upon DUX withdrawal. Knocking down of *Zfp352* reversely delayed the down-regulation of MT2_Mm and 2C transcription factors at various time points during the exit from DUX-induced 2CL state (Supplementary Fig. 2I), potentially because 2C genes were already up-regulated upon *Zfp352* knockdown before release. Besides, in the *Dux* transgene-independent TBLC state (Supplementary Fig. 2J), the overexpression of *Zfp352* during the release from TBLC state by PlaB withdrawal (Supplementary Fig. 2H) also promoted the exit from MERVL positive state (Fig. 1F), and MT2_Mm, as well as 2C transcription factors were also down-regulated faster (Fig. 1G). Collectively, these data signify a role of ZFP352 in destabilizing the in vitro totipotency network and promoting 2C state dissolution, despite of being a top enriched transcription factor in the mouse 2C embryo.

### ZFP352 activates post-2C stage genes in mouse embryonic development

**Fig. 2:**
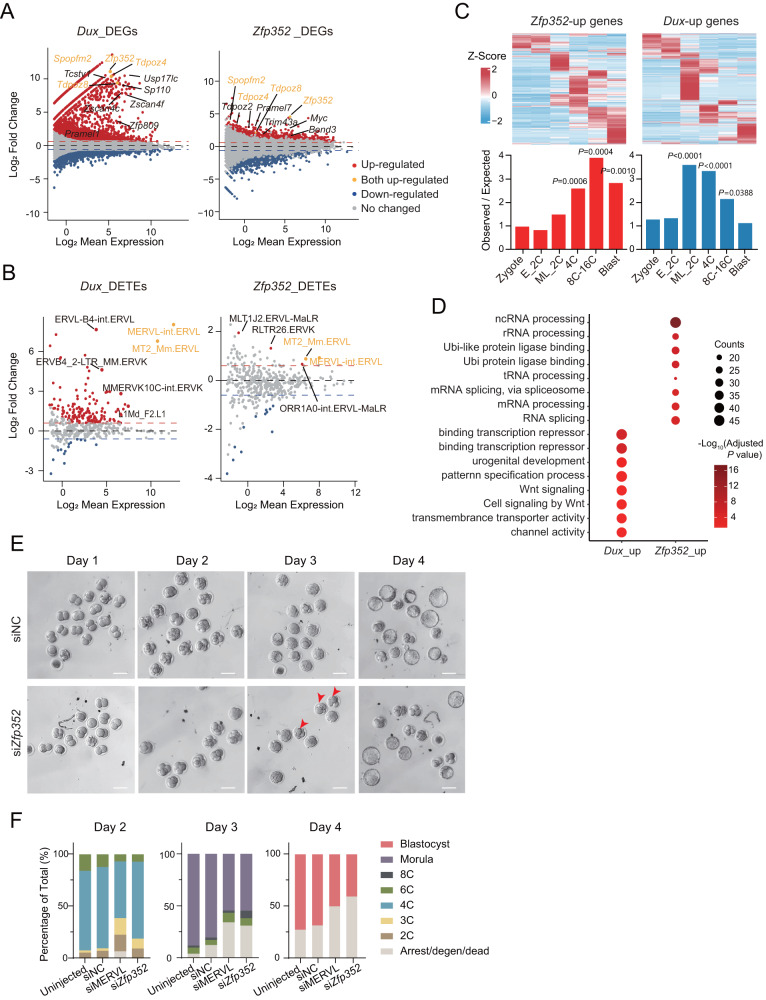
Transcriptome changes induced by ZFP352. **A**, **B** MA plot showing the log_2_ expression fold change and log_2_ mean expression upon overexpression of *Zfp352* (12 h) and *Dux* (24 h) in mESCs as measured by RNA-seq (duplicates) for genes (**A**) or transposable elements (TEs) (**B**). The red and blue dots indicate up- or down-regulated genes or TEs (fold change [FC] > 1.5/<−1.5 and Adjusted *P* value < 0.05), and the orange dots indicate the commonly up-regulated genes or TEs upon *Zfp352* and *Dux* overexpression (two-sided Fisher’s exact test, multiple test by FDR method). **C** The embryo stage specific expression dynamics for ZFP352 or DUX-induced DEGs. The top heatmaps showing the Z-score of gene expression changes at different embryonic stages; the bottom barplots showing the relative enrichment for ZFP352 or DUX-induced DEGs at different embryonic stages. The relative enrichment was calculated by the observed over expected number of DEGs (one-sided hypergeometric test, multiple test by Benjamini & Hochberg method, E_2C: early 2C, ML_2C: mid_late 2C, Blast: blastocyst). **D** The GO analysis for ZFP352 and DUX up-regulated genes. The size of the dots represents gene counts enriched in the terms and the color code represents the –log_10_(Adjusted *P* value) (one-sided hypergeometric test and multiple test by Benjamini & Hochberg method). **E**, **F** Mouse embryos at zygotic stage were injected with siRNA targeting MERVL or *Zfp352*, uninjected embryos or embryos injected with scrambled siRNA (siNC) were used as control. The embryos development was followed for four days upon injection to analyze the effect of siRNA treatment. The embryo morphology from day 1 to day 4 after injection was shown in the bright field pictures in (**E**), and the red arrows indicating the embryos with delayed development observed at day 3 after si*Zfp352* injection (scale bar = 100 μm); the percentage of embryos reaching different developmental stages at day 2, day 3 and day 4 was shown in the barplot in (**F**). The total numbers of embryos in each group of treatment were as follows: uninjected embryos (*n* = 51), siNC embryos (*n* = 41), siMERVL (*n* = 44), si*Zfp352* (*n* = 42).

To explore the potential function of ZFP352 in mESCs and mouse early embryos, firstly we exogenously expressed *Zfp352* in mESCs from a dox-inducible promoter (Supplementary Fig. 2A). Overexpression of *Zfp352* barely resulted in the activation of MERVL fluorescent reporter driven by MT2_Mm sequence, as well as the endogenous MT2_Mm, compared to that induced by DUX (Supplementary Fig. 3B, C), suggesting ZFP352 did not play a significant role in promoting 2C state. To reveal the transcription mechanism underlying the phenotypes of ZFP352, transcriptome changes upon ZFP352 overexpression were profiled, and compared with the transcription effect elicited by DUX. Notably, ZFP352 induced largely different genes and retrotransposons compared to DUX (Fig. 2A, B, Supplementary Data 2 and Supplementary Data 3). Only a few 2C-specific genes, like *Tdpoz* and *Spopfm* family genes, were shared between *Zfp352* and *Dux* overexpression (Fig. 2A, Supplementary Fig. 3D). Instead, ZFP352 up-regulated DEGs were enriched with 4C to blastocyst specific genes (Fig. 2C and Supplementary Fig. 3E, F); while on the other hand, DUX up-regulated genes were more significantly clustered around mid_late 2C to 4C stages (Fig. 2C and Supplementary Fig. 3E, F). GO analysis revealed that DUX-induced genes were mainly involved in transcription and signaling, while ZFP352-induced genes were enriched in pathways related to RNA and protein metabolism (Fig. 2D). The phenotypic and transcriptomic distinction of ZFP352 compared to DUX, implies a potential “post-2C” developmental function by ZFP352.

To explore the function of ZFP352 during mouse early embryonic development, siRNA targeting *Zfp352* was injected into the zygotes and the effect of *Zfp352* depletion on development was followed (Supplementary Fig. 3G, H). siRNA for MERVL was used as a control as the depletion of which was previously shown to impair blastocyst formation and delay the embryo development from 2C to 4C stage (Supplementary Fig. 3G, I)^51^. Similar to knocking down of MERVL, siRNA targeting *Zfp352* reduced the blastocyst formation rates (Fig. 2E, F). However, while MERVL depletion led to delayed transition from 2C to 4C embryos at day 2, *Zfp352* knocking down did not result in obvious delay in 4C embryo formation at day 2; instead, reduced entry into morula stage at day 3 was observed (Fig. 2E red arrows, and 2F). Further transcriptome profiling of *Zfp352* siRNA treated embryos from day 2 to day 4 revealed that although si*Zfp352* embryos largely progressed into 4C and later stages, mid-late 2C and 4C specific genes remained highly expressed, while 8C and later developmental genes failed to be switched on properly (Supplementary Fig. 3J, K). The later developmental delay and failure to down-regulate 2C genes caused by *Zfp352* depletion further substantiate an impact of ZFP352 in embryogenesis after 2C.

### ZFP352 binds on both MT2_Mm and SINE_B1/Alu elements

**Fig. 3:**
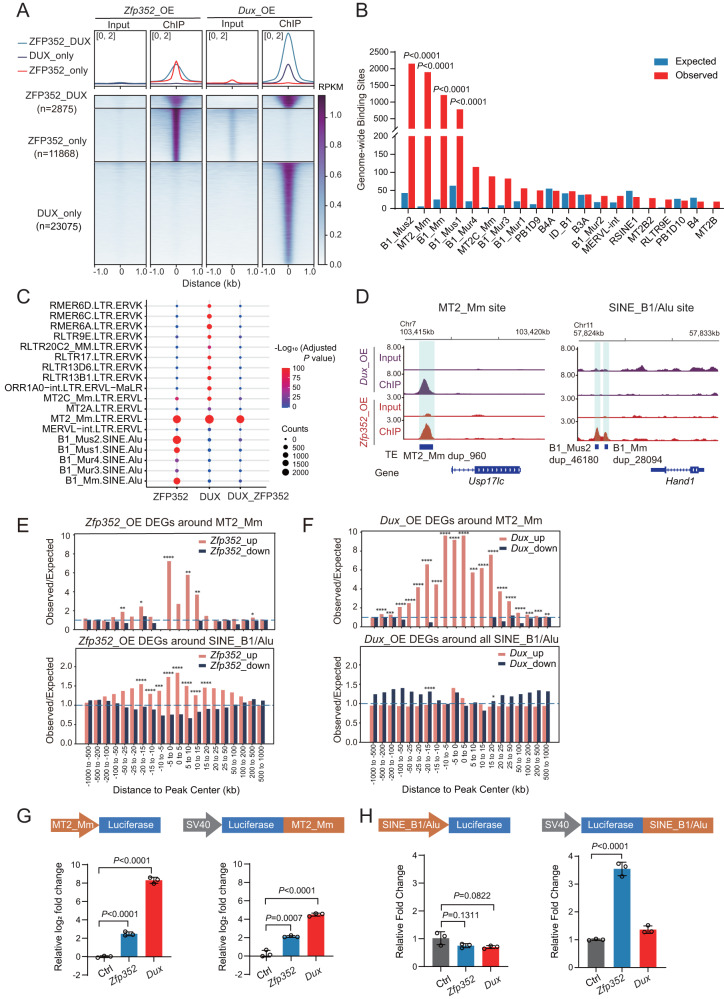
ZFP352 and DUX have both overlapping and distinct binding sites. **A** Metaplots and heatmaps of ZFP352 and DUX ChIP-seq (GSE95517) signal over ZFP352_DUX overlapping peak center (blue), ZFP352_only peak center (red), and DUX_only peak center (purple). ZFP352 or DUX ChIP-seq were done in *Zfp352* or *Dux* over-expressed mESCs, respectively. **B** Barplot showing the genome-wide binding sites for the top 20 TE sub-families enriched in the ZFP352 ChIP-seq peaks. The y-axis indicates the expected and observed numbers of ZFP352_bound retrotransposon sub-families (one-sided hypergeometric test, multiple test by Benjamini & Hochberg method). **C** Enrichment analysis showing TE sub-families with significant overlap with ZFP352 peaks, DUX peaks and ZFP352_DUX overlapping peaks. The size of the dots represents the counts of overlapped peaks and the color code represents the significance of the overlap shown by −log_10_(Adjusted *P* value) (one-sided hypergeometric test, multiple test by Benjamini & Hochberg method; value greater than 100 was all labeled as 100). **D** Coverage plot of DUX (purple) and ZFP352 (red) ChIP-seq signals over selected MT2_Mm (dup_960) and nearby gene *Usp17lc* (left), selected SINE-B1/Alu (dup_46180, dup_28094) and nearby gene *Hand1* (right). **E**, **F** Barplot representing the association of DEGs induced by *Zfp352* overexpression (**E**) and *Dux* overexpression (**F**) with genome-wide MT2_Mm and SINE_B1/Alu loci analyzed by RAD. **G** Luciferase assay measuring the activation effect of ZFP352 and DUX on MT2_Mm driven luciferase reporter. The barplots showing the relative log_2_(fold change) of luciferase gene expression upon *Zfp352* or *Dux* overexpression in HEK293T cells. The graphs showing MT2_Mm consensus sequence cloned as a promoter or enhancer for luciferase gene expression (mean ± SD, *n* = 3 biologically independent samples, two-sided unpaired t-test). **H** Luciferase assay measuring the activation effect of ZFP352 and DUX on SINE_B1/Alu driven luciferase reporter. The barplots showing the relative fold change of luciferase gene expression upon *Zfp352* or *Dux* overexpression in HEK293T cells. The graphs showing SINE_B1/Alu (dup_35706) cloned as a promoter or enhancer for luciferase gene expression (mean ± SD, *n* = 3 biologically independent samples, two-sided unpaired t-test).

To further understand the mechanism underlying ZFP352 function, and how ZFP352 and DUX regulate distinct sets of genes with different developmental dynamics, genome-wide binding sites of ZFP352 were profiled by ChIP-seq and compared with the binding pattern of DUX. ZFP352 and DUX shared 2875 co-bound sites, while majority of binding peaks were distinct between ZFP352 and DUX^8^ (Fig. 3A). Region-associated DEGs analysis (RAD)^47^ was performed to assess the enrichment of ZFP352 or DUX-induced DEGs around different types of ChIP-seq binding sites (Supplementary Fig. 4A, B). ZFP352-induced DEGs were enriched nearby ZFP352_DUX common peaks and ZFP352_only peaks (Supplementary Fig. 4A); while DUX-induced DEGs were highly enriched nearby ZFP352_DUX common peaks and DUX_only peaks (Supplementary Fig. 4B). ZFP352 ChIP-seq peaks largely overlapped with retrotransposons sequences (Supplementary Fig. 4C), especially the SINE and LTR classes (Supplementary Fig. 4D). ZFP352 bound retrotransposons were also significantly associated with ZFP352-induced DEGs in the nearby regions (Supplementary Fig. 4E). Comparing different retrotransposons sub-families, MT2_Mm, B1_Mus2, B1_Mm, B1_Mus1 were the top-enriched ones bound by ZFP352 (Fig. 3B). Among them, MT2_Mm, the LTR for MERVL, was found to be significantly shared between ZFP352 and DUX ChIP-seq peaks (Fig. 3C and Supplementary Fig. 4F). A closer examination of the ChIP-seq tracks confirmed the co-localization of ZFP352 and DUX binding sites on MT2_Mm sequences (Fig. 3D and Supplementary Fig. 4G). On the contrary, ZFP352 bound SINE_B1/Alu sequences were absent from DUX binding, while DUX preferentially bound to ERVK sub-family LTRs (Fig. 3C, D and Supplementary Fig. 4F, G), indicating ZFP352 and DUX transcriptionally target distinct retrotransposons sub-families.

To investigate whether the binding of ZFP352 and DUX on different retrotransposons accounts for their diverged transcriptional function in 2C state, the enrichment of ZFP352 or DUX DEGs (Fig. 2A, C) in the neighboring regions of MT2_Mm or SINE_B1/Alu elements was assessed by RAD analysis (Fig. 3E, F). ZFP352 up-regulated DEGs were statistically enriched nearby both MT2_Mm and SINE_B1/Alu elements (Fig. 3E and Supplementary Fig. 4H), while the number of DEGs nearby SINE_B1/Alu were more (Supplementary Fig. 4I). In contrast, DUX up-regulated DEGs were only enriched nearby MT2_Mm elements (Fig. 3F). The preferential distribution of ZFP352 DEGs, but not DUX DEGs, nearby SINE_B1/Alu implies that ZFP352 may utilize SINE_B1/Alu elements to activate distinct subsets of genes compared to DUX.

To further assess whether MT2_Mm or SINE_B1/Alu DNA sequences can be directly bound and activated by ZFP352 or DUX, selected MT2_Mm or SINE_B1/Alu genomic sequences were cloned into the promoter or enhancer based luciferase reporter constructs (Fig. 3G, H). Ectopic expression of *Dux* or *Zfp352* both activated MT2_Mm to drive downstream luciferase expression, although the extent of MT2_Mm reporter activation was much weaker for ZFP352 (Fig. 3G). While on the other hand, ZFP352, but not DUX, activated SINE_B1/Alu as an enhancer to switch on luciferase expression (Fig. 3H). This further validates that ZFP352 and DUX preferentially activate distinct retrotransposon sub-families. In addition, it is interesting to note that MT2_Mm can function either as promoter or enhancer to activate luciferase expression (Fig. 3G), while SINE_B1/Alu can only function as enhancer to do so (Fig. 3H). Correspondingly, *Zfp352* overexpression increased genome-wide accessibility of SINE_B1 loci (Supplementary Fig. 4J), although up-regulation of SINE_B1 RNA expression did not co-occur (Supplementary Fig. 4K). Such a decoupling of SINE_B1 chromatin openness and expression level was also evident during the early embryonic development^46,52^ (Supplementary Fig. 4L, M).

### ZFP352 activates SINE_B1/Alu genes to promote 2C state dissolution

**Fig. 4:**
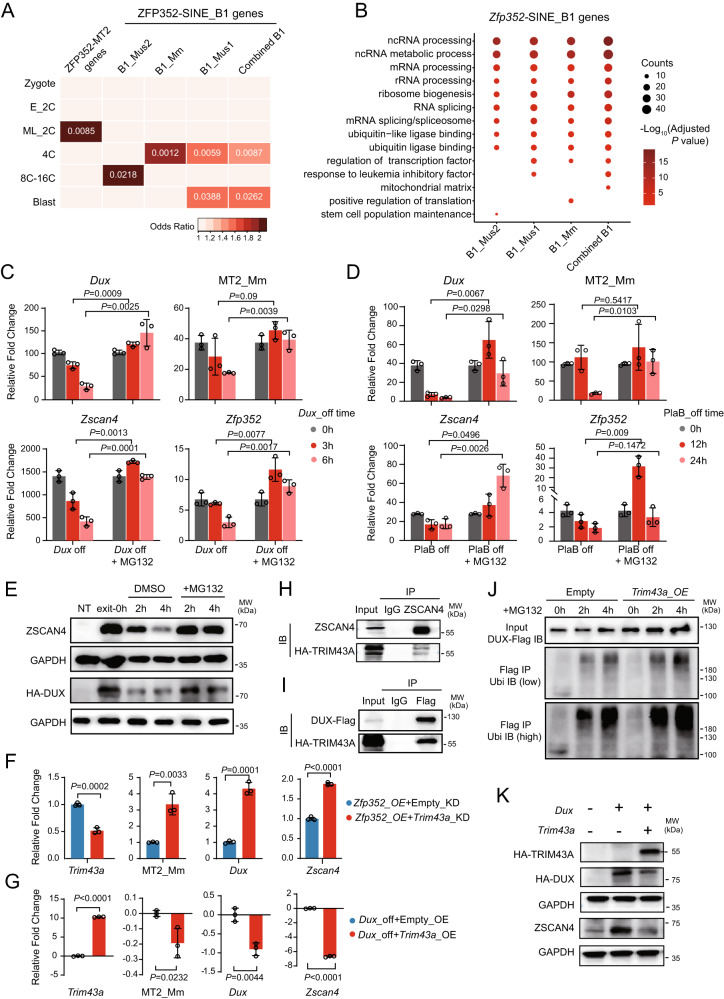
ZFP352 targeted SINE_B1/Alu genes are involved in destabilization of 2C state. **A** Enrichment analysis for the overlap of ZFP352-MT2 genes and different types of ZFP352-SINE_B1 genes with the developmental stage marker genes in mouse embryo. Color code represents the odd ratio of enrichment, and numbers represent Adjusted *P* value of enrichment (E_2C: early 2C, ML_2C: mid_late 2C, Blast: blastocyst). **B** GO analysis for different types of ZFP352 up-regulated SINE_B1 nearby genes. The size of the dots represents gene counts enriched in the respective terms and the color code represents –log_10_(Adjusted *P* value). **C**, **D** Barplots showing the effect of proteasome inhibition (+MG132) on key 2C marker expression measured by RT-qPCR during the exit from 2CLC (**C**) or TBLC (**D**) states. In **C**, the exogenously-expressed *Dux* was switched off to allow the exit from 2CL state for 3 h or 6 h with MG132 added in this process. In **D**, PlaB was removed for 12 h and 24 h to allow exit from TBLC state with MG132 added 6 h prior to RNA collection. **E** Western blot showing the effect of MG132 treatment on 2C protein stability during the exit from DUX-induced 2CL state. ZSCAN4 and HA-DUX protein level at 0 h, 2 h and 4 h of cyclohexamide (CHX) addition were shown. **F**, **G** Barplots showing the relative expression changes of 2C genes upon *Trim43a* knocking down (**F**) or overexpression (**G**) in mESCs over-expressing *Zfp352*. **H**, **I** Co-immunoprecipitation of TRIM43A and ZSCAN4 (**H**), or TRIM43A and DUX-Flag (**I**) in HEK293T cells over-expressing HA-*Trim43a* and *Zscan4*, or HA-*Trim43a* and *Dux*-Flag. ZSCAN4 or DUX-Flag was pulled down to detect the co-precipitation of TRIM43A. **J** Western blot showing the ubiquitination of DUX by TRIM43A. *Dux*-Flag was over-expressed in HEK293T cells with or without *Trim43a* overexpression. DUX-Flag was pulled down to detect the ubiquitination level. **K** Western blot showing the effect of *Trim43a* overexpression on 2C proteins. HA-*Trim43a* and HA-*Dux* were over-expressed in DUX-induced 2CLCs. **A** and **B** used one-sided hypergeometric test and multiple test by Benjamini & Hochberg method; **C**, **D**, **F**, and **G** were presented as mean ± SD, *n* = 3 biologically independent samples, two-sided unpaired t-test.

Both ERVs and SINE can potentially act as cis-elements to regulate nearby gene transcription^33,40,41,53^. While MT2_Mm was closely involved in the 2C transcription network^37^, the effect of SINE_B1/Alu during early embryogenesis was not clearly defined. The ZFP352 up-regulated DEGs nearby ZFP352 bound SINE_B1/Alu sites (ZFP352-SINE_B1 genes) expressed mostly at or after 4C stage (Fig. 4A and Supplementary Fig. 5A). The temporal expression patterns of ZFP352-SINE_B1 genes during embryonic development suggest that ZFP352 may confer post-2C developmental function through binding and activation of SINE_B1/Alu genes.

GO analysis of up-regulated ZFP352-SINE_B1 genes revealed RNA splicing, rRNA processing, and ubiquitination as significantly up-regulated pathways (Fig. 4B). Notably, both RNA splicing (*Isy1* etc.) and rRNA synthesis (*Lin28a* etc.) related genes have recently been implicated in antagonizing 2C state^17–19,54^. While whether ubiquitination and protein degradation play a role in the dissolution of 2CL state was not known. We firstly evaluated the potential effect of ubiquitination pathway in 2C exit by blocking the E1 ubiquitin-activating enzyme (using TAK243), proteasome (using MG132), or lysosome (using Chloroquine) function, respectively (Supplementary Fig. 5B, C). During the exit from DUX-induced 2CL state, inhibiting E1 or proteasome activity led to extended expression of MT2_Mm and 2C genes (Fig. 4C; Supplementary Fig. 5D); lysosome inhibition also had a similar but milder effect (Supplementary Fig. 5E). In addition, during the exit from TBLC state upon withdrawal of PlaB, E1 or proteasome inhibition also led to delayed down-regulation of MT2_Mm and 2C genes, while lysosome inhibition was less effective in delaying the exit from TBLC state (Fig. 4D and Supplementary Fig. 5F, G). During the exit from 2CL state, key 2C transcription factors, like ZSCAN4 and DUX, became more stabilized upon proteasome inhibition (Fig. 4E), potentially contributing to the delayed dissolution of 2CL state upon MG132 treatment. These data collectively suggest that ubiquitination-mediated protein degradation can promote the dissolution of 2C state, similar as the effect of RNA splicing and rRNA synthesis pathways as previously reported^17–19^. Thus, ZFP352 may transcriptionally evoke multiple 2C antagonizing pathways through SINE_B1/Alu to facilitate the dissolution of totipotent state.

Among the ubiquitination and proteasome-related SINE_B1/Alu genes significantly up-regulated by ZFP352 (Supplementary Fig. 5H), many of them were also up-regulated from 4C to blastocyst stages (Supplementary Fig. 5I). To further dissect the molecular mechanism for ZFP352 activated ubiquitination genes in 2C state dissolution, we focused on TRIM43A, one of the E3 ubiquitination ligases activated by ZFP352 through B1_Mus1 and B1_Mm elements (Supplementary Figs. 4G, 5H), and highly enriched from 4C to 16C stage embryos (Supplementary Fig. 5I). Knocking down of *Trim43a* in *Zfp352* over-expressing cells, resulted in increased MT2_Mm and 2C genes expression (Fig. 4F). Conversely, the overexpression of *Trim43a* resulted in decreased expression of MT2_Mm and 2C genes during the exit from DUX-induced 2CL state (Fig. 4G), suggesting TRIM43A plays important roles during the dissolution of 2C state. TRIM43A can bind directly to 2C proteins, like ZSCAN4 and DUX (Fig. 4H, I and Supplementary Fig. 5J), and it can also directly ubiquitinate ZSCAN4 and DUX (Fig. 4J and Supplementary Fig. 5K). Overexpression of *Trim43a* led to reduced abundance of 2C proteins, like DUX and ZSCAN4 (Fig. 4K), but it did not result in reduction of pluripotent transcription factor OCT4 (Supplementary Fig. 5L). Collectively, these data implies that TRIM43A can antagonize 2C state through destabilization of 2C transcription factors. Hence, ubiquitination pathway genes induced by ZFP352 can facilitate the dissolution of 2C state through regulating the stability of 2C proteins.

### ZFP352 binding shift from MT2_Mm to SINE_B1 facilitates totipotency dissolution

So far, the data delineated differential binding of ZFP352 towards two distinct retrotransposons, MT2_Mm and SINE_B1/Alu. While MT2_Mm is typically associated with 2C maintenance, we showed that the binding of ZFP352 onto SINE_B1/Alu and activation of nearby genes can act to promote the dissolution of totipotent state. More interestingly, using CRISPR activation or inactivation system targeting MT2_Mm sequences (Supplementary Fig. 6A, B) led to reversed regulatory effect on ZFP352-SINE_B1 genes expression (Supplementary Fig. 6C, D), further highlighting an inversely correlation between these two retrotransposons. Thus this promoted us to look for the mechanism leading to the differential binding and regulatory effect by ZFP352 on MT2_Mm and SINE_B1.

**Fig. 5:**
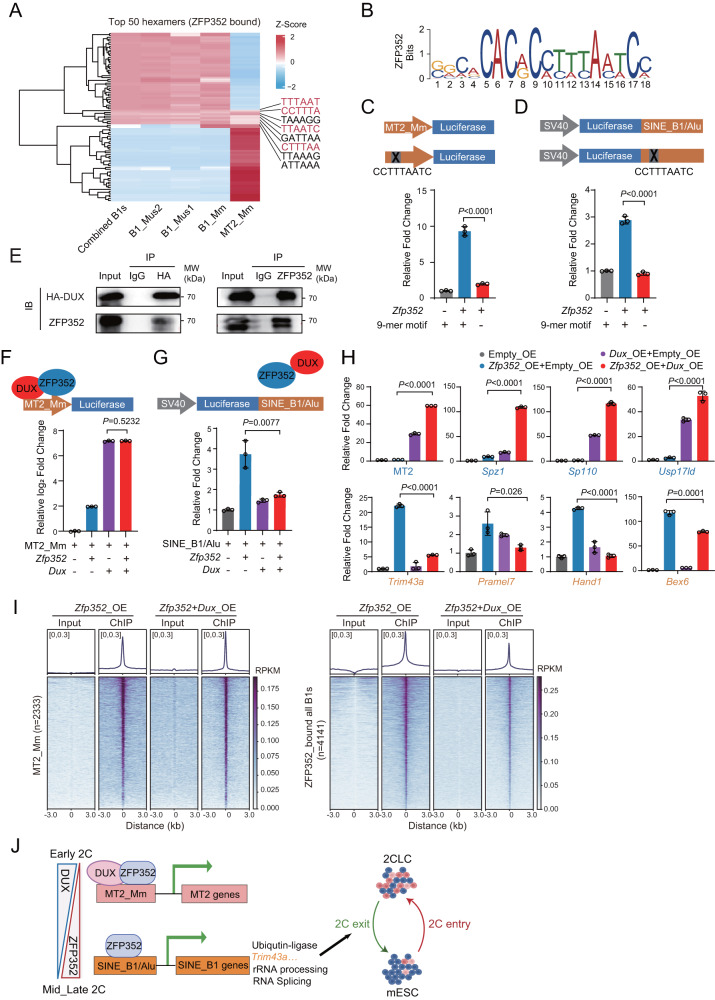
Differential regulation of MT2_Mm and SINE_B1/Alu by ZFP352. **A** Heatmap showing the abundance of the respective top 50 enriched hexamers from ZFP352 bound SINE_B1 sequences and MT2_Mm sequences in different types of retrotransposon sub-family. The color codes represent Z-score for the enrichment of a hexamer. The hexamer sequences significantly enriched in both ZFP352 bound SINE_B1 and MT2_Mm were labeled. **B** The top enriched de novo motif identified from the ZFP352 ChIP-seq peaks using MEME. **C** Luciferase assay measuring the effect of ZFP352 in activating the luciferase reporter driven by MT2_Mm or MT2_Mm with the deletion of the consensus 9-mer motif in HEK293T cells. **D** Luciferase assay measuring the effect of ZFP352 in activating the luciferase reporter driven by SINE_B1/Alu or SINE_B1/Alu with the deletion of the consensus 9-mer motif in HEK293T cells. **E** Co-immunoprecipitation of ZFP352 and DUX in mESCs (E14) over-expressing HA-*Dux* and *Zfp352*. HA and ZFP352 antibodies were used for pulldown and detection of the co-precipitated DUX and ZFP352 protein, respectively. **F**, **G** Luciferase assay measuring the single effect and coordinated effect of ZFP352 and DUX in activating luciferase reporters driven by MT2_Mm (**F**) and SINE_B1/Alu (**G**) in HEK293T cells. The barplots showing the relative expression fold change of luciferase genes. **H** Barplots showing the single effect and coordinated effect of ZFP352 and DUX on MT2_Mm and SINE_B1 gene expression in mESCs. The relative fold change of MT2_Mm, nearby 2C genes (*Sp110*, *Usp17ld*, *Spz1*), SINE_B1/Alu nearby genes (*Trim43a*, *Hand1*, *Bex6, Pramel7*) were measured by RT-qPCR and normalized to mESCs with empty plasmid overexpression. **I** Metaplots and heatmaps of ZFP352 ChIP-seq signal over MT2_Mm regions, ZFP352 bound SINE_B1 regions. ZFP352 ChIP-seq were performed in *Zfp352* over-expressing or *Zfp352* and *Dux* co-overexpressing mESCs, respectively. **J** Summary and working model for ZFP352’s function during the 2C dissolution process. Created with BioRender.com. Barplots in **C**, **D**, **F**, **G**, and **H** were presented as mean ± SD, *n* = 3 biologically independent samples, two-sided unpaired t-test.

Firstly, we try to identify if ZFP352 binds to MT2_Mm and SINE_B1/Alu through different sequence features. The enrichment scores of random hexamers were calculated for ZFP352 bound MT2_Mm and ZFP352 bound SINE_B1/Alu sequences, respectively. Among the top 50 hexamers, respectively, enriched in MT2_Mm or SINE_B1 sequences, eight hexamers were shared between MT2_Mm and SINE_B1/Alu (Fig. 5A and Supplementary Data 4). Interestingly, four of them exist as continuous nine base-pair motif, CCTTTAATC, which was also present in the top predicted motif from ZFP352 ChIP-seq peaks (Fig. 5A, B). When this motif was deleted in the luciferase reporters for MT2_Mm or SINE_B1/Alu, luciferase induction by ZFP352 was completely abolished (Fig. 5C, D). This implies that the binding of ZFP352 onto both MT2_Mm and SINE_B1/Alu were mediated through the same sequence motif.

Next, we tried to identify whether the binding choices on MT2_Mm or SINE_B1/Alu by ZFP352 were mediated through other transcription partners. ZFP352 and DUX can co-bind onto MT2_Mm (Fig. 3C, D). Interestingly, immunoprecipitation experiment demonstrated that ZFP352 and DUX interacted with each other in both mESCs (Fig. 5E) and HEK293T cells (Supplementary Fig. 6E). Although co-expression of ZFP352 sustained the activation of the MT2_Mm luciferase reporter by DUX in HEK293T cells (Fig. 5F), the co-existence of DUX led to near-complete abolishment of the SINE_B1/Alu luciferase activation by ZFP352 (Fig. 5G). This implies that the interaction between ZFP352 and DUX is permissive to MT2_Mm activation but restricts SINE_B1 activation. Furthermore, in mESCs although ZFP352 alone has little activation towards 2C genes, it exhibited synergistic effect with DUX to further boost subsets of 2C genes induced by DUX (Fig. 5H); on the other hand, the induction of SINE_B1/Alu genes by ZFP352 was severely impaired by the presence of DUX (Fig. 5H). Correspondingly, further ChIP-seq experiments validated that overexpression of *Dux* displaced ZFP352 from SINE_B1/Alu and DUX-independent ZFP352 binding sites, but supported ZFP352 binding onto MT2_Mm and DUX_ZFP352 co-bound sites (Fig. 5I and Supplementary Fig. 6F). In vitro ELISA assay similarly showed reduced binding on SINE_B1 probe by ZFP352 in the presence of DUX (Supplementary Fig. 6G). These data collectively suggest a model whereby ZFP352 potentially interacts with DUX to bind MT2_Mm; while on its own, ZFP352 binds and activates SINE_B1/Alu and nearby genes.

During early embryonic development, the activation of ZFP352 bound MT2_Mm genes precedes the activation of ZFP352 bound SINE_B1/Alu genes (Fig. 4A). To see whether this is also associated with different dynamics of ZFP352 and DUX in mouse embryos, we assessed their expression pattern in the pre-implantation mouse embryo single cells RNA-seq and Ribo-seq data^46,55^. For both RNA expression and protein translation dynamics, *Dux* expressed earlier than *Zfp352* (Supplementary Fig. 6H, I), and was found to be the upstream regulator of *Zfp352* (Fig. 2A and Supplementary Fig. 6J). Thus in early and mid 2C embryos, ZFP352 was activated by DUX^56^ (Fig. 2A and Supplementary Fig. 6J, K), and during the initial activation stage, ZFP352 may first coordinate with high level of DUX to bind MT2_Mm (Fig. 5I). In late 2C and 4C stage, ZFP352 level continued to rise while DUX expression gradually declined^46,55^ (Supplementary Fig. 6H, I), creating a window period when excess free ZFP352 can bind to SINE_B1/Alu (Fig. 5I) and initiate the expression of 2C dissolution genes (Fig. 5H). This is consistent with our initial observation that further overexpression of *Zfp352* in DUX-induced 2CLCs promoted 2C exit (Fig. 1D–G). Furthermore, in mouse embryos, knocking down of *Zfp352* by siRNA also led to impaired transition from 4C to morula stage, a later developmental defect compared to MERVL knocking down (Fig. 2E, F, and Supplementary Fig. 3J, K). These data signify that the selective binding of ZFP352 onto MT2_Mm and SINE_B1/Alu may be temporally programmed and segregated during embryogenesis, and the shifted binding of ZFP352 from MT2_Mm towards SINE_B1/Alu promotes a timely and coordinated transition out of the totipotent state (Fig. 5J).

## Discussion

The studies employing the in vitro totipotent-like models emerged from pluripotent stem cells have advanced our understanding of the totipotency regulatory network. Transcription factors like DUX and ZSCAN4 families are previously identified to promote 2C-specific gene expression and enforce the totipotency transcription network^8,9,28^. On the other hand, antagonizing factors of totipotent state have also been identified, the inhibition of which promoted the reversion of pluripotent ESCs back to 2CL state^15,17,18^. Potentially, 2C antagonizing factors are also implicated in the dissolution of the totipotent state^19,45^. However, from the totipotent state, how the antagonizing machineries are triggered to initiate the transition out of the 2C embryo or 2CL state was not clear. The evidence revealed from this study suggests that the transition out from the 2CL state, or potentially 2C embryos, is intrinsically wired in the totipotency transcription network, such that the 2C specific and highly expressed transcription factors, like ZFP352, can facilitate a programmed 2C dissolution (Fig. 5J).

During early embryonic development, maternal and early 2C transcription factors, like DUX, activate other 2C genes, including *Zfp352* (Fig. 2A and Supplementary Fig. 1E). We found that upon activation, ZFP352 can physically interact and coordinate with DUX to bind on MT2_Mm sites (Figs. 3D–F, 5F, H, I and Supplementary Fig. 4F, G). Although ZFP352 alone has minimal activation towards 2C genes, its coordinated binding with DUX boosted subsets of 2C-specific genes induced by DUX (Figs. 2A and 5H). Aside from boosting 2C genes, the binding of ZFP352 with DUX, may also serve to sequester ZFP352 from untimely binding and activation of SINE_B1/Alu and 2C antagonizing genes in the early 2C stage. Only with continuous accumulation of ZFP352 and concurrent reduction of DUX from late 2C to 4C stage (Supplementary Fig. 6H, I), a tiling point can be triggered where ZFP352 abundance surpassed DUX and DUX-free ZFP352 became dominant. Free ZFP352 can then activate 2C antagonizing genes (Figs. 4A, B and 5H), promoting the dissolution of 2C state (Fig. 5J). However, a limitation in our current study is that we have not been able to pin-point the relative amount of ZFP352 over DUX to elicit SINE_B1/Alu binding and 2C exit effect, and more quantitative experimental system is needed for measuring this.

Interestingly in this process, ZFP352, as a highly expressed 2C transcription factor (Supplementary Fig. 1E), appears to exhibit selective binding on two apparently “contradicting” retrotransposons, MT2_Mm and SINE_B1/Alu. While MT2_Mm was known to be important in the establishment and maintenance of 2C state^37^, we found in this study that, upon binding onto SINE_B1/Alu, ZFP352 can increase their chromatin accessibly (Supplementary Fig. 4J) and activate nearby genes to promote the totipotency dissolution (Fig. 4 and Supplementary Fig. 5). The binding of ZFP352 onto SINE_B1s did not led to SINE_B1 RNA expression changes (Supplementary Fig. 4K). Thus, the up-regulation of SINE_B1 RNA during the mid-late 2C stage of mouse embryonic development (Supplementary Fig. 4L) may instead be induced by other transcription factors than ZFP352. Moreover, the SINE_B1 expression was maintained relatively constant, independent of the chromatin accessibility states in early mouse embryo (Supplementary Fig. 4M). Furthermore, we show that the differential targeting preference towards MT2_Mm or SINE_B1/Alu by ZFP352 was influenced by the co-presence of DUX (Figs. 3D–F, 5F–I, and Supplementary Fig. 6F, G). This potentially ensures the triggering of totipotency exit by SINE_B1/Alu genes is kept minimal until ZFP352 is in over-abundance with DUX in late 2C to 4C stage.

Notably, a few molecular pathways downstream of the ZFP352 bound SINE_B1/Alu were shown to facilitate the dissolution of totipotent state, including RNA splicing, rRNA biogenesis, and ubiquitination. While recent studies highlighted the 2C antagonizing effects of RNA splicing and rRNA biogenesis pathways^17,18^, the effect of protein ubiquitination on the totipotency regulation has not been reported. Previous studies shows that protein ubiquitination is involved in switching off the pluripotency-related proteins and necessary for efficient differentiation of ESCs^3^. Similarly, during the exit from the totipotency network, rather than waiting for gradual and passive dilution of totipotency proteins, we find that cells mobilize active ubiquitination and protein degradation machineries to destabilize and eliminate totipotency transcription factors, and promote the dissolution of totipotent state (Fig. 4 and Supplementary Fig. 5). Thus, ZFP352 may initiate multiple pathways to modulate RNA and protein homeostasis for efficiently silencing of the totipotency network.

Overall, our study hereby showcases how two distinct retrotransposons are selectively bound by the same transcription factor, and the shifted binding of ZFP352 from MT2_Mm to SINE_B1/Alu programmed cell fate switching during early embryogenesis. Retrotransposons are tightly associated with early embryogenesis in both mouse and human, and they exhibit high activities and specificities in different embryonic stages. Yet, their functional roles as well as their dynamic regulatory mechanisms in early embryos remain largely elusive. Our study employing in vitro totipotency models and mouse embryos shed insights on retrotransposon regulation and functions during early development. It also potentiates different angles for dissecting the unexplained pre-implantation developmental failure in clinical conditions.

## Methods

### Cell culture and cell lines

HEK293T cells (Pricella, CL-0005) were cultured on 0.12% gelatin pre-coated dishes with DMEM high glucose (HyClone, SH30243.01) supplemented with 10% FBS (Gibco, 10270-106). E14 cells (Cell Search System, E14tg2a) were cultured in DMEM high glucose (Gibco, 11965-084) with 15% FBS (Gibco, 16000-044), 100X NEAA (Gibco, 11140050), 100X L-Glutamine (Gibco, 25030081), 1000X β-Mercaptoethanol (Gibco, 21985023), homemade LIF (10,000X dilution) on gelatin pre-coated plate. The medium was refreshed daily. E14 cells were passaged by incubation with 0.05% trypsin (Beyotime, C0203) for 3 min at 37 °C. ZFP352 inducible mESC line was made by lentiviral integration of the pCW57.1-*Zfp352* expression plasmid. *Zfp352* coding sequence was cloned under the TET inducible promoter in the pCW57.1 plasmid. Cells were routinely checked for mycoplasma contamination.

### Transfection and transduction

Transfection was done using Lipofectamine 2000 (Life Technologies, 11668027) according to protocol. Transfection mix was added to the gelatin pre-coated plate, before cells were seeded. For lentivirus production, HEK293T cells were grown to 70% confluency. The transfer plasmid, pxPAX2, and VSVG were all transfected into HEK293T cells with PEI (Polysciences, 24765-1). Supernatant was harvested 48 h to 72 h later, and virus produced was precipitated using 5X virus concentration solution (Origene, TR30026), resuspended in PBS, and aliquoted before storing at −80 °C. To infect cells with lentivirus, virus was added into the medium with 4 μg/ml polybrene for 24 h. Medium was refreshed the second day to allow the subsequent assays.

### RNA extraction and RT-qPCR

Total RNA was extracted by RNAios Plus (Takara, 9109). RNA was reverse-transcribed into cDNA by HiScript II Q RT SuperMix (Vazyme, R223-01). qPCR was performed using ChamQ SYBR qPCR Master Mix (Vazyme, Q311-03) with the Roche LightCycler® 480 (LC480) system.

### Luciferase expression assay

The SINE_B1 sequences and MT2_Mm sequence were cloned into pGL4.23 luciferase reporter plasmid containing the SV40 mini-promoter, or the psi-CHECK-2 (Promega, C8021) luciferase reporter plasmid. Luciferase reporter plasmids were transfected into HEK293T cells together with *Zfp352* or *Dux* over-expressing plasmids. The luciferase expression changes were detected by RT-qPCR. Luciferase activity was also measured with the dual luciferase assay system (Promega, E1910) and analyzed on a Synergy2 plate reader (BioTek).

### Co-immunoprecipitation

Cells were lysed in Endo-IP buffer with protease inhibitor cocktail (Biotool, B14001) on ice for 30 min, and the lysate was centrifugated at 4 °C for 25 min to remove precipitates. 500 μg total protein was incubated with 5 μg of rabbit IgG or HA antibodies (Sigma, H3663) or Flag antibodies (Sigma, F1804) at 4 °C overnight with rotation. Lysate and antibody mix was incubated with 30 μl protein G Dynabeads (Thermo Fisher Scientific, 10003D) for 2 h at room temperature with rotation. Beads were washed with lysis buffer and eluted. The eluent was analyzed by western blotting. To detect protein ubiquitination, 500 μg total protein was incubated with 5 μg of rabbit IgG or ZSCAN4 antibodies (Millipore, AB3430) at 4 °C overnight with rotation. Lysate and antibody mix were incubated with 30 μl protein G Dynabeads (Thermo Fisher Scientific, 10003D) for 2 h at room temperature with rotation. The eluent was later blotted with ubiquitin antibody (CST, #3936).

### Western blotting

Cell lysate in Endo-IP buffer with protease inhibitor cocktail or Co-IP eluted samples were denatured at 95 °C and kept on ice. Protein samples were resolved using SDS-PAGE and transferred onto a polyvinylidene difluoride (PVDF) membrane (Sigma-Aldrich, 3010040001) using a wet tank transfer system. The PVDF membrane was blocked in 10% non-fat milk in PBST for 1 h and incubated the primary antibody in blocking buffer at 4 °C overnight and subsequently secondary antibody conjugated with HRP (Genescrip, A00098) 1:5000 diluted. The membrane was visualized using the Azure C300 system. The primary antibody used included antibody against GAPDH (Abclonal, AC033), HA (Sigma, H3663), Flag (Sigma, F1804), ZSCAN4 (Millipore, AB3430), ubiquitin (CST, #3936) and ZFP352, and all primary antibodies were diluted at 1:2000.

### Flow cytometry

mESCs were digested into single cells by 0.05% trypsin at 37 °C and fixed with 4% PFA for 30 min. Upon resuspended in pre-cold PBS, the suspensions were filtered through 40 µm strainer and analyzed on BD LSRFortessa^TM^ Cell Analyser. The FACS data were analyzed using FlowJo X V10, with gating strategies illustrated in Supplementary Fig. 7.

### CRISPRi and CRISPRa

For the CRISRi and CRISPRa system, sgRNAs designed from MT2_Mm consensus sequences in Dfam were used and cloned into dCAS9-VP160 and dCAS9-KRAB vectors modified from Addgene #48240. The sgRNA sequences used in this study were:

CAGCTGTGAATGGAAGTCCA

ATTTATTGATGACTTACAGT

CACCAGTGACCCTTATCTGG

dCAS9-VP160 or dCAS9-KRAB plasmid with sgRNAs were transduced into E14 cells and empty vectors were used as control.

### Embryo retrieval and microinjection

The 8–10-week-old female ICR mice were purchased from Shanghai SLAC Laboratory Animal Co., Ltd. All the mice were housed under the SPF environment with a 12 h light-dark cycle, and had a temperature of 22–24 °C with 50–60% humidity. ICR female mice (8–12 weeks old) were super ovulated by i.p. injection of 10 IU pregnant mare’s serum gonadotropin (PMSG, CEN’S, Hangzhou, China) and 48 h later, 10 IU human chorionic gonadotropin (hCG, CEN’S, Hangzhou, China). For in vivo produced embryos, females were then mated with ICR male mice (10–18 weeks old). The zygotes were collected from dissected oviducts at 0.5 dpc. and put into KSOM medium (ARK Resource Co., Ltd.). Zhejiang University (China) provided the guidance for the animal research protocol with the ethical approval number as ZJU20230182.

For siRNA injections, the in vivo collected zygotes were randomly allocated into four groups: si*Zfp352*, siMERVL, siNC (scrambled control), and uninjected control. The siRNA sequences were as shown below.

si*Zfp352*: CCAUUUGAGAACACUUCUUTT; GCUCCAUAUGUGGGUGAAUTT; GGUUCUACGCUUGUCCCUUTT

siMERVL: GAAGAUAUGCCUUUCACCAGCUCUA

siNC: UUCUCCGAACGUGUCACGUTT

60 μM of siRNA and were injected into the cytoplasm of zygotes using microinjection glass capillary (BOROSILICATE GLASS, ITEM#: BF100-78-15). Injected embryos were cultured in KSOM medium under mineral oil in 35 mm petri dishes (Corning Life Sciences, 430165) in the humidified multi-gas incubator (5% O_2_, 6% CO_2_, and 89% N_2_) at 37 °C. Embryo development were recorded for a total of four days after microinjection.

### Real-time qPCR and RNA-seq for mouse embryos

Total RNA was isolated and pooled from 10 embryos. cDNA conversion was performed as previously described^57^. Embryos were obtained and placed into a 0.2-ml thin-walled PCR tube containing 2 µl of cell lysis buffer (with 5% RNase inhibitor and 95% Triton X-100), 1 µl of 10 µM oligo-dT primer and 1 µl of dNTP mix (10 mM each; Fermentas, R0192). The mix was quickly vortexed and then span down at 700 × *g* for 10 s at room temperature) and immediately transferred on ice. The samples were incubated at 72 °C for 3 min and immediately put back on ice. 5.7 µl of the RT mix containing SuperScript II reverse transcriptase (Invitrogen, cat. no. 18064-014), RNAse inhibitor (10U), 5X Superscript II first-strand buffer, 100 mM DTT (Invitrogen, 18064-014), 5 M Betaine (BioUltra ≥99.0%; Sigma-Aldrich, 61962), 1 M MgCl_2_, 100 µM TSO were mixed by gently pipetting up and down. The sample was incubated at 42 °C for reverse transcription, and then amplified with First-strand reaction KAPA HiFi HotStart ReadyMix (KAPA Biosystems, KK2601). The relative expression levels of *Zfp352* and MERVL were measured by RT-qPCR and normalized to *Gapdh*. The cDNA was used for library construction using Vazyme TruePrep DNA Library Prep Kit V2 for Illumina kit (TD503-01) and subsequently subjected to Illumina platform sequencing with a depth of 20 M reads per sample. More detail about library construction can be found in its user’s manual.

### ELISA assay

SINE_B1 DNA probe was synthesized by PCR using biotinylated primer, diluted in 0.1% TBS-T, and coated onto streptavidin plates (Thermo Scientific, 15500). The probe-coated wells were washed and blocked before loaded with different amounts of protein extract from HEK293T cells over-expressing *Zfp352* or *Zfp352*+*Dux* together. Primary antibody against HA at 1:2000 dilution ratio, and anti-mouse secondary antibody coupled with HRP at 1:5000 dilution ratio was used to detect HA-ZFP352 bound to SINE_B1 probe. The wells were further incubated with OPD-solution (Sangon Biotech A610348) in the dark for 30 min before adding the stop solution. The emission was measured at 492 nm with 650 nm as reference wavelength.

### Repeat elements annotation

We downloaded RepeatMasker (rmsk) tracks of mm10 from UCSC genome browser (https://genome.ucsc.edu/). We further filtered TEs with large indels, which are those with length 20% longer or shorter than its consensus sequences (annotated in Repbase). We then generated a new gene annotation file in GTF format by combining filtered TEs with Ensembl gene annotation (version GRCm38.99). The combined GTF file was used in RNA-seq or Chip-seq analysis subsequently.

### Single-cell RNA-seq analysis

#### Re-processing mouse early embryo development scRNA-seq data

In order to quantify the expression of TEs during early embryonic development, we re-processed scRNA-seq data (SMART-Seq) from^46^ as previously described^58^. Briefly, the raw files were downloaded from short reads achieve (SRA) database (accession: SRA072494) and mapped to mouse reference genome GRCm38.99 with STAR (v2.7.0e)^59^. Reads mapped to not more than 2000 loci were retained, and only the best hit was kept (–alignEndsType EndToEnd –winAnchorMultimapmax 2000 –outFilterMultimapNmax 2000 –outSAMprimaryFlag AllBestScore –outSAMmultNmax 1). With such setting, all uniquely mapped reads will be kept. For reads with multiple equivalent best hits of equivalent scores, only one of the hits will be retained. We then used featureCounts (v2.0.0)^60^ to quantify the number of reads mapped to both gene and TE with parameter (-s 0 –fraction -M -C).

The count matrix generated by featureCounts was loaded into Seurat (v3.0.0)^61^ for downstream processing, including basic QC, normalization, clustering. We kept cells that expressed over 2000 gene features and filtered out cells with 50% of their RNAs derived from mitochondrial. We then normalized raw counts to counts per 10k reads and identified the top 3000 most variable genes with Seurat function “NormalizeData”, “FindVariableFeatures” and “vst” selection method. The variable genes were scaled and used as input to compute the Principal Components (PCs). Top 50 PCs were selected for computing Uniform Manifold Approximation and Projection (UMAP) with Seurat function “RunUMAP” using default settings. The cell type information was adopted from ref. ^46^. Stage specific marker genes were identified with Seurat function “FindMarkers” and parameters: “min.pct = 0.2, logfc.threshold = 1, test.use = “wilcox”, max.cells.per.ident = 20”.

The cell type (embryonic stage of cell) marker gene list was defined according to the annotation file from ref. ^46^. Markers were identified using FindMarker function in Seurat. Average FC > 2 and *P* value < 0.05 were used as cut-off for defining the final markers, and the marker gene list was used for the following analysis.

#### Integrative analysis of 2CLC entry and exit process using scRNA-seq data

For the integrative analysis of 2CLC entry and exit process, we downloaded raw fastq files from a study of entry 2CLC induced by *Dux* overexpression^15^ (GEO: GSE121459) and from a study of exit from DUX-induced 2CLC^45^ (GEO: GSE133234). Since these datasets are generated from 10X Genomics platform, we first processed it by STARsolo with the following parameters: (--winAnchorMultimapNmax 2000 --outFilterMultimapNmax 2000 --outSAMprimaryFlag AllBestScore --outSAMmultNmax 1 --limitOutSJoneRead 10000 --limitOutSJcollapsed 3000000 --outSAMattributes NH HI nM AS CR UR CB UB GX GN sS sQ sM --soloType CB_UMI_Simple --soloCBwhitelist 737K-april-2014_rc.txt --soloCBlen 14 --soloUMIstart 15 --soloUMIlen 8 --soloBarcodeReadLength 0 --soloStrand Forward --soloFeatures Gene GeneFull SJ). In addition, the combined GTF, in which TE annotation has been added, was used for quantifying the TEs abundance. Using such settings, we identified cells expressed MT2_Mm or other TEs.

Using the count matrix generated from STARSolo, downstream analysis was done in Seurat. Briefly, we excluded cells with (1) less than 400 UMI/cell and (2) less than 500 or more than 8000 genes detected, and with more than 30% of UMIs derived from mitochondrial RNAs. Raw UMI counts were normalized to counts per 10k UMIs and log-transformed. Top 3000 most variable genes were identified with Seurat function “FindVariableFeatures” and variable selection method “vst” for both datasets, respectively. We then applied “FindIntegrationAnchors” and “IntegrateData” functions to the top 20 CCAs to integrate the two datasets. UMAP of integrated datasets was computed by the “RunUMAP” function with setting “n.neighbors = 5”.

### RNA-seq and data analysis

The library was constructed with Truseq RNA Sample Prep Kit v2 (Illumina, RS-122-2001) before being subjected to Illumina platform sequencing with a depth of 20 M reads per sample. Quality control for raw reads was performed by FastQC (v0.11.8)^62^. The first 10 bp of both paired-end reads were trimmed by cutadapt (v2.9)^63^. STAR (v2.7.0e)^59^ was used for aligning reads against mouse reference genome GRCm38.99 (https://ftp.ensembl.org/pub/release-99/fasta/mus_musculus/dna/). Reads with no more than 2000 mapped loci were retained, and only the best hit was kept (–alignEndsType EndToEnd –winAnchorMultimapmax 2000 –outFilterMultimapNmax 2000 –outSAMprimaryFlag AllBestScore –outSAMmultNmax). Quantification for both gene and TE were calculated by FeatureCounts v2.0.0^60^ with parameter using GRCm38.99 GTF file(-s 0 –fraction -M –C, https://ftp.ensembl.org/pub/release-99/gtf/mus_musculus/).

Differentially expressed genes (DEGs) and transposable elements (DE-TEs) were generated by the R package edgeR (v3.30.3)^64^. Only features with mean TPM greater than 1 in either control or treatment group were retained. DEGs were defined as fold change > 2 and Adjusted *P* value < 0.05. GO term enrichment for DEGs was conducted with the R package clusterProfiler (v3.12.0)^65^ (ont = ‘ALL’, pAdjustMethod = ‘BH’, pvalueCutoff = 0.05, qvalueCutoff = 0.05).

The clustering marker gene lists were defined with genes match the following criterion. Firstly the DEGs were filtered out at several different time point after Dux overexpression, and then DEGs with the highest TPM compared with other different time points and also higher than the average TPM of the other time points were defined as *Dux* overexpression time point clustering specific genes. Overlapping analysis was performed with the R package GeneOverlap (v1.30.0, GitHub - shenlab-sinai/GeneOverlap: R package for testing and visualizing gene list overlaps).

### ChIP-seq and data analysis

Cells were harvested and fixed with 1% formaldehyde (Sigma, 47608-250ML-F) for 10 min at RT with rotation. Quenching was done with 0.14 M glycine at room temperature for 10 min. Cells were lysed by ChIP lysis buffer (10 mM Tris-HCl (pH 8.0), 0.25% Triton X-100, 10 mM EDTA, 100 mM NaCl, protease inhibitor cocktail). The genomic DNA was sonicated into short fragments with an average size of 500 bp. The fragmented DNA was incubated with 3 μg HA (CST, 61099) antibody overnight at 4 °C. The mix was subsequently incubated with 30 μl protein G Dynabeads for 2 h at room temperature with rotation. DNA was eluted in elution buffer (50 mM Tris-HCl (pH 8.0), 1 mM EDTA, 1% SDS), treated with proteinase K at 60 °C overnight. DNA was purified by FastPure DNA Extraction Mini Kit (Vazyme Biotech Co. Ltd, DC301) and subjected to qPCR or Illumina sequencing.

We used a community-curated bioinformatics pipelines “nf-core/chipseq” (v1.1.0)^66^ for the analysis of ChIP-seq data. We took the default setting of the chipseq pipeline but added the “--keep-multi-map” option to retain multiple hits reads so that peaks in TE region can be identified. Briefly, raw fastq files were first fitered by FastQC (v0.11.8)^62^. TrimGalore (v0.5.0, https://www.bioinformatics.babraham.ac.uk/projects/trim_galore/) was used for trimming adapters. The remaining high-quality reads were mapped to mouse reference genome downloaded from Ensembl (GRCm38.99) by BWA (v0.7.17)^67^. PCR duplicates were marked by PICARD (v2.19.0, http://broadinstitute.github.io/picard/) and removed by SAMtools (v1.9)^68^. Then, MACS2 (v2.1.2)^69^ was used for calling peaks. The narrow peaks with q-value (minimum FDR) < 0.05 were kept as final peaks.

Bigwig tracks were generated using Deeptools (v3.4.3)^70^ by normalizing to RPKM using binsize of 10 bp. ChIP-seq signals over genomic regions were plotted by Deeptools (v3.4.3)^70^. For TE annotation of peak region, annotation of different TE families/classes over peak regions (Observed) was obtained by annotatePeaks.pl function in Homer (v4.7)^71^ by using TE annotation file.

*Dux* overexpression ChIP-seq raw data were from GEO: GSE95517^8^. ChIP-seq heatmap were plotted using computeMatrix and plotHeatmap commands from DeepTools (v3.4.3)^70^. K-means clustering method was used to divide the regions into three distinct categories. The resulting clusters are ZFP352 and DUX co-binding region (±500 bp), DUX only binding region without ZFP352 and ZFP352 only binding region without DUX. The Genome browser images of peak regions and read coverage were composed using the Integrative Genomics Viewer (IGV). Peaks were annotated against mm10 with annotation library from UCSC.

The expected TE locations surrounding ZFP352 ChIP peaks were identified by counting the number of the respective TE sub-family copies in binding regions of the ZFP352 ChIP peak region, and the expected TE numbers were computed assuming random distribution of TE sub-family in the respective genomic region. The average length of each TE sub-family was taken into account as well. SINE_B1 specific genes and B1_Mus2, B1_Mus1, B1_Mm specific genes were counted using the BEDtools (v2.29.2)^72,73^ window command in a window of ±5 kb from the summits of the peaks. MT2_Mm specific genes were counted using the BEDtools window command in a window of ±50 kb from the summits of the peaks because of the total copy numbers of SINE_B1 were much higher than the numbers of MT2_Mm, so different distance criterion was used.

### ATAC-seq and data analysis

ATAC-seq was performed as previously described^74^ using MagicSeq Tn5 DNA Library Prep Kit for Illumina (Magic-Bio, M3141). mESC pellet was treated transposase at 37 °C for 30 min, purified using MinElute PCR Purification Kit (QIAGEN, 28006), and amplified using 1xNEBnext PCR master mix (NEB, M0541S) using custom Nextera PCR primers 1 and 2. Upon purification with MinElute PCR Purification Kit (QIAGEN, 28006). Libraries were subjected to Nova pair-end 150 bp sequencing.

For ATAC-seq data analysis, quality control was performed by FastQC v0.11.8^62^. Tn5 adapter (AGATGTGTATAAGAGACAG) was trimmed by cutadapt v2.9^63^. STAR v2.7.0e^59^ was used for alignment onto human reference genome GRCm38.99. Reads with maximal 1000 multiple mapped sites and no more than 3 mismatches were retained, and only the best hit was kept (--outFilterMultimapNmax 1000, --outFilterMismatchNmax 3, --outSAMmultNmax 1). By creating STAR index without general feature format file and not allowing intron length (--alignIntronMax 1), the splice junction was neglected. PCR duplicates were removed by Samtools v1.2^68^ rmdup function.

ATAC-seq peaks were defined using MACS2 v2.2.7.1^75^ callpeaks function with default parameters. Bigwig tracks were generated using deeptools v3.4.3^70^ by normalizing to RPKM using binsize of 10 bp. ATAC-seq signals over genomic regions were plotted by deeptools v3.2.1^70^.

### Motif analysis

ZFP352 bound SINE_B1 sequences were SINE_B1/Alu peaks which have ZFP352 ChIP-seq peaks in ±5 kb region (*n* = 4141). ZFP352 bound B1_Mus2 (*n* = 2150), ZFP352 bound B1_Mus1 (*n* = 780) and ZFP352 bound B1_Mm (*n* = 1211) were also obtained with the same method. ZFP352 bound MT2_Mm sequences were MT2_Mm peaks which have ZFP352 ChIP-seq peaks in ±50 kb region (*n* = 1896). Motif analysis was done by Motif Discovery function from the MEME web site using ZFP352 bound SINE_B1 and ZFP352 bound MT2_Mm bed files. Hexamer analysis was conducted using ZFP352 bound SINE_B1 or ZFP352 bound MT2_Mm sequences. The sequences information of ZFP352 bound SINE_B1 or ZFP352 bound MT2_Mm were generated using the getfasta command from BEDtools (v2.29.2)^72,73^ with their respective bed file, and the hexamers were counted using Jellyfish (v2.3.0)^76^ count command, and Jellyfish dump command was used to obtain the statistics from the hexamer count results. The percentage distribution of every hexamer in each ZFP352_bound TE sub-family was calculated as the percentage of this hexamer among all random hexamers from the retrotransposons sub-family copies and plotted together in the heatmap.

### Data visualization

Heatmaps of selected genes was plotted using R package pheatmap (v1.0.12) and ComplexHeatmap (v2.0.0)^77^.

### RAD analysis

The RAD analysis was done using the web tool: https://labw.org/rad/docs^47^. The DEGs from *Dux* overexpression and *Zfp352* overexpression RNA-seq were selected with the following criteria: up-regulated genes with fold change [FC] > 1.5 and Adjusted *P* value < 0.05; down-regulated genes with fold change [FC] < −1.5 and Adjusted *P* value < 0.05. The input regions include the DUX or ZFP352 bound peaks revealed from ChIP-seq, MT2_Mm, and SINE_B1 sub-family location coordinates in the GRCm38 (mm10) genome. For submit options, “GRCm38(mm10)” was selected as reference genome, “1000, 500, 200, 100, 50, 25, 20, 15, 10, 5, 0 kb” as the customized peak extend distance. The enrichment scores were calculated by observed over expected distribution frequencies. “Expected” represents the genomic average assuming random distribution. One-sided hypergeometric test was performed to evaluate the statistical significance, and the *P* value were presented as the following: *P < 0.05, **P < 0.01, ***P < 0.001, ****P < 0.0001.

### Statistics and reproducibility

For all the data presented in the figures except for data from Next Generation Sequencing, the experiments were performed at least twice, each time with replicates and statistical analysis were as described in the respective figure legends.

### Reporting summary

Further information on research design is available in the Nature Portfolio Reporting Summary linked to this article.

## Supplementary information

Supplementary Information

Description of Additional Supplementary Files

Supplementary Data 1

Supplementary Data 2

Supplementary Data 3

Supplementary Data 4

Supplementary Data 5

Reporting Summary

## Data Availability

The RNA-seq, ATAC-seq, ChIP-seq, and embryo RNA-seq data generated in this study have been deposited at GEO database under the accession code GSE222636. The raw data for the barplot and micrographs generated in this study are provided in source data file. The gel blots are provided in the Supplementary Figures (Supplementary Fig. 8 and Supplementary Fig. 9). Primers used in the manuscript are available in Supplementary Data 5. Data referenced in this study are available in Gene Expression Omnibus (GEO) with the references and accession numbers: DUX CHIP data (GEO: GSE95517), Mouse embryo scRNA-seq (GEO: GSE45719), *Dux*_OE 2C-enrty scRNA-seq (GEO: GSE121459), *Dux*_OE 2C-exit scRNA-seq (GEO: GSE133234), Mouse embryo ATAC-seq (GEO: GSE66390), Mouse embryo Ribo-seq (GEO: GSE165782), and Mouse embryo *Dux*_KO RNA-seq (GEO: GSE121746). Source data are provided with this paper.

## Source data

Source Data

## Code availability

The codes of bioinformatics analysis have been deposited at https://github.com/zhengyilii/ZFP352_2C/tree/Code_Data (10.5281/zenodo.8000606) and are publicly available as of the date of publication. Any additional information required to re-analyze the data reported in this paper is available from the lead contact upon request.
