# Supplementary material for: Selective binding of retrotransposons by ZFP352 facilitates the timely dissolution of totipotency network
Source: Nat Commun. 2023 Jun 20;14:3646. doi: 10.1038/s41467-023-39344-1 (PMC10281998; doi:10.1038/s41467-023-39344-1)
Supplement: Supplementary file 1 — Supplementary Information [file 41467_2023_39344_MOESM1_ESM.pdf]

Supplementary information

Selective binding of retrotransposons by ZFP352 facilitates the timely dissolution of totipotency network

Zhengyi Li, Haiyan Xu, Jiaqun Li, Xiao Xu, Junjiao Wang, Danya Wu, Jiateng Zhang, Juan Liu, Ziwei Xue, Guankai Zhan, Bobby Cheng Peow Tan, Di Chen, Yun-Shen Chan, Huck Hui Ng, Wanlu Liu, Chih-Hung Hsu, Dan Zhang, Yang Shen, Hongqing Liang

Supplementary Figure 1-9

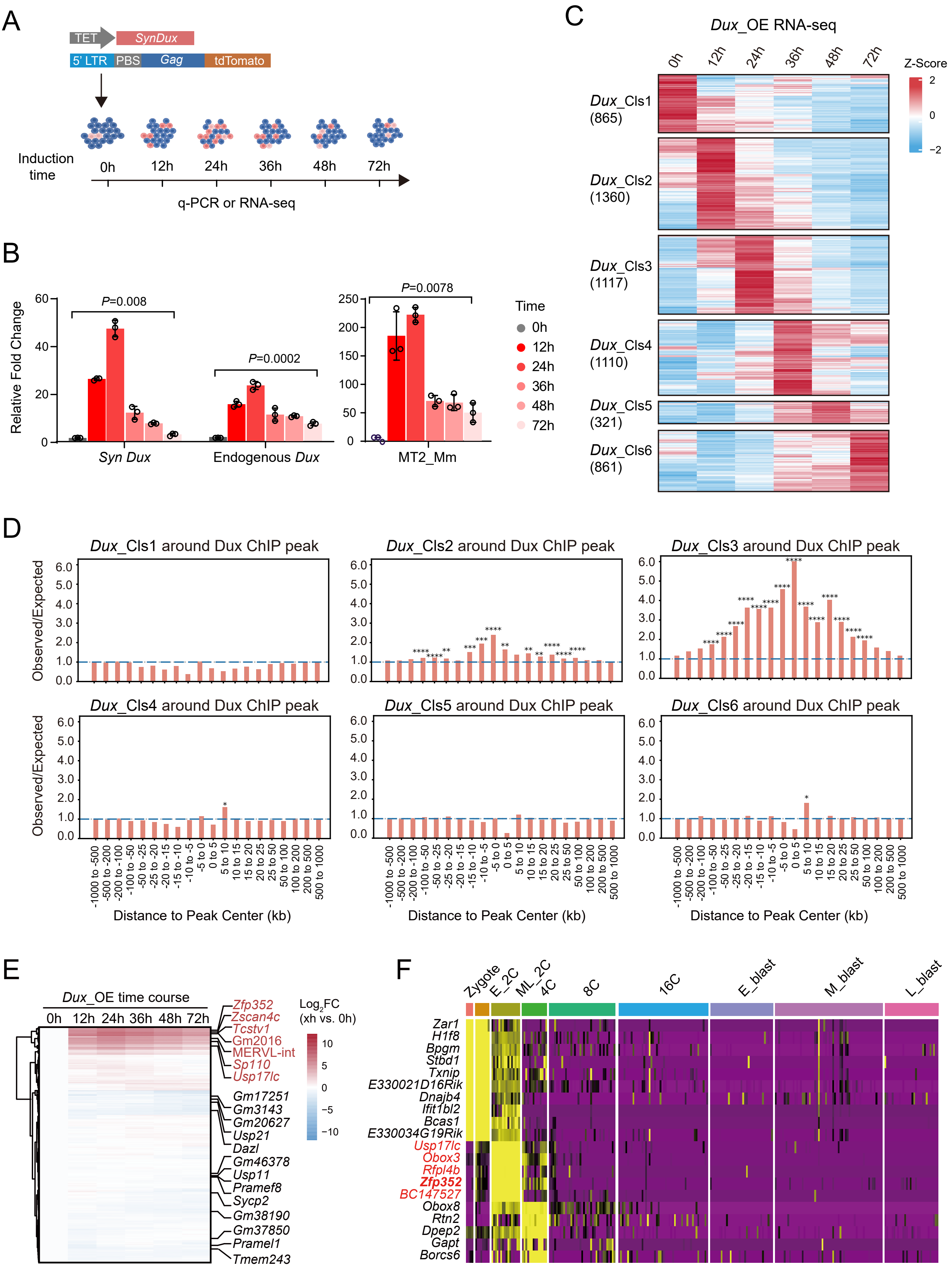

A
TET
SynDux
5' LTR
PBS
Gag
tdTomato
Induction time
0h
12h
24h
36h
48h
72h
q-PCR or RNA-seq
B
Relative Fold Change
P=0.008
P=0.0002
P=0.0078
Syn Dux
Endogenous Dux
MT2_Mm
Time
0h
12h
24h
36h
48h
72h
C
Dux_OE RNA-seq
Z-Score
Dux_Cls1 (865)
Dux_Cls2 (1360)
Dux_Cls3 (1117)
Dux_Cls4 (1110)
Dux_Cls5 (321)
Dux_Cls6 (861)
D
Dux_Cls1 around Dux ChIP peak
Dux_Cls2 around Dux ChIP peak
Dux_Cls3 around Dux ChIP peak
Dux_Cls4 around Dux ChIP peak
Dux_Cls5 around Dux ChIP peak
Dux_Cls6 around Dux ChIP peak
Observed/Expected
Distance to Peak Center (kb)
E
Dux_OE time course
0h 12h 24h 36h 48h 72h
Log2FC (xh vs. 0h)
Zfp352
Zscan4c
Tcstv1
Gm2016
MERVL-int
Sp110
Usp17lc
Gm17251
Gm3143
Gm20627
Usp21
Dazl
Gm46378
Usp11
Pramef8
Sycp2
Gm38190
Gm37850
Pramel1
Tmem243
F
Zygote
E_2C
ML_2C
4C
8C
16C
E_blast
M_blast
L_blast
Zar1
H1f8
Bpgm
Stbd1
Txnip
E330021D16Rik
Dnajb4
Ifit1bl2
Bcas1
E330034G19Rik
Usp17lc
Obox3
Rfpl4b
Zfp352
BC147527
Obox8
Rtn2
Dpep2
Gapt
Borcs6

Supplementary Figure 1: 2C gene expression dynamics in DUX induced 2CLCs.

A) Schematics illustrating the inducible *Dux* over-expression cell line and experimental design for transcriptome profiling. *Dux* sequence was codon-optimized and denoted as syn*Dux*. Created with BioRender.com.

B) The relative expression level of exogenously-expressed *Dux* (syn*Dux*), endogenous *Dux* and MT2_Mm upon *Dux* over-expression for 0h,12h, 24h, 36h, 48h and 72h as measured by RT-qPCR (mean ± SD, n = 3 independent samples, two-sided unpaired t-test).

C) Heatmap showing the Z-scores for the DEGs at different time points in the *Dux* over-expression RNA-seq (two replicates); DEGs were grouped into six clusters based on their average expression pattern at different time points upon *Dux* induction, and the number of DEGs in every cluster was indicated.

D) The barplot showing the association between DEGs in different *Dux*-OE clusters and the DUX ChIP-seq peak center analyzed by RAD.

E) Heatmap showing the expression dynamics of mid_late 2C specific genes after *Dux* induction for different time points. The expression was normalized to un-induced mESCs at 0h. The color code represents $\log_2$(xh vs. 0h).

F) Heatmap showing the Z-scores of top five stage-specific protein coding genes from scRNA-seq of early mouse embryos (GSE45719); cells from the same developmental stages were clustered in column under the same color. The top enriched genes in mid_late 2C were highlight in red. (E_2C: early 2C, ML_2C: mid_late 2C, E_Blast, M_Blast or L-Blast: early, mid or late blastocyst).

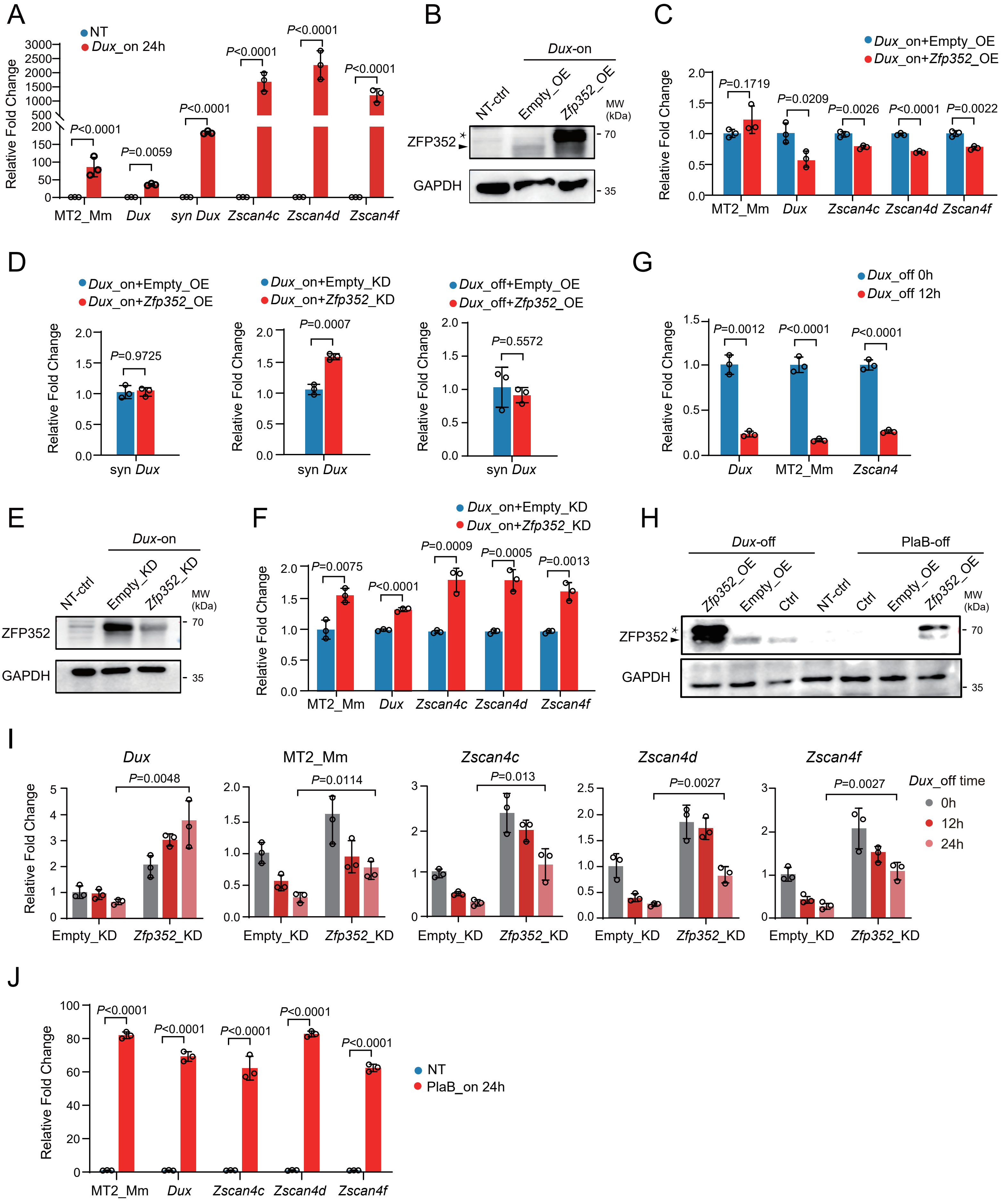
A
NT
Dux_on 24h
Relative Fold Change
P<0.0001
P=0.0059
MT2_Mm
Dux
syn Dux
Zscan4c
Zscan4d
Zscan4f
B
Dux-on
NT-ctrl
Empty_OE
Zfp352_OE
MW (kDa)
ZFP352
GAPDH
70
35
C
Dux_on+Empty_OE
Dux_on+Zfp352_OE
P=0.1719
P=0.0209
P=0.0026
P<0.0001
P=0.0022
D
Dux_on+Empty_OE
Dux_on+Zfp352_OE
P=0.9725
Dux_on+Empty_KD
Dux_on+Zfp352_KD
P=0.0007
Dux_off+Empty_OE
Dux_off+Zfp352_OE
P=0.5572
G
Dux_off 0h
Dux_off 12h
P=0.0012
P<0.0001
Zscan4
E
Dux-on
NT-ctrl
Empty_KD
Zfp352_KD
F
Dux_on+Empty_KD
Dux_on+Zfp352_KD
P=0.0075
P<0.0001
P=0.0009
P=0.0005
P=0.0013
H
Dux-off
PlaB-off
Zfp352_OE
Empty_OE
Ctrl
NT-ctrl
I
P=0.0048
P=0.0114
P=0.013
P=0.0027
Dux_off time
0h
12h
24h
Empty_KD
Zfp352_KD
J
NT
PlaB_on 24h

Supplementary Figure 2: Effect of ZFP352 during the exit from 2CL state.
A) The relative expression level of 2C genes in DUX induced 2CLC (24h induction) measured by RT-qPCR.
B) Western blot showing ZFP352 protein level when over-expressed in DUX induced 2CLCs. Empty_OE or *Zfp352*_OE: over-expression of empty or *Zfp352* plasmid in 2CLCs for 12h. (arrow: endogenous ZFP352, asterisk: exogenous HA-ZFP352)
C) The relative expression changes of key 2C markers upon *Zfp352* over-expression in 2CLCs for 12h .
D) The relative expression level of exogenous *Dux* upon over-expressing *Zfp352* for 12h or knocking down *Zfp352* for 24h in 2CLCs, or during exit from 2CLCs for 12h.
E) Western blot showing ZFP352 protein level upon *Zfp352* knocking-down in 2CLCs. Empty_KD or *Zfp352*_KD: shRNA knocking down non-targeting or *Zfp352* sequences in 2CLCs (arrow: endogenous ZFP352, asterisk: exogenous HA-ZFP352, ML_2C: mid_late 2C, 8C-Blast: 8C-blastocyst).
F) The relative expression changes of key endogenous 2C markers upon knocking down *Zfp352* by shRNA in 2CLCs.
G) The relative expression level of 2C markers upon 12h of doxycycline withdrawal to switch off exogenous *Dux* expression.
H) Western blot showing ZFP352 protein level in experiments from Fig.1D, E and 1F, G. NT-ctrl: no treatment control; *Dux*-off or PlaB-off represents the treatment used to release from 2CLC or TBLC state; Ctrl: no other treatment upon *Dux*-off or PlaB-off treatment; Empty_OE or *Zfp352*_OE: over-expression of empty or *Zfp352* plasmid during exit for 12h.
I) The relative expression changes of key endogenous 2C markers upon *Zfp352* knocking down at 12h and 24h after releasing from 2CLCs by doxycycline withdrawal .
J) The relative expression level of 2C markers in PlaB induced TBLC (24h treatment).
(A), (C), (D), (F), (G), (I) and (J) presented as mean ± SD, n = 3 biologically independent samples, two-sided unpaired t-test.

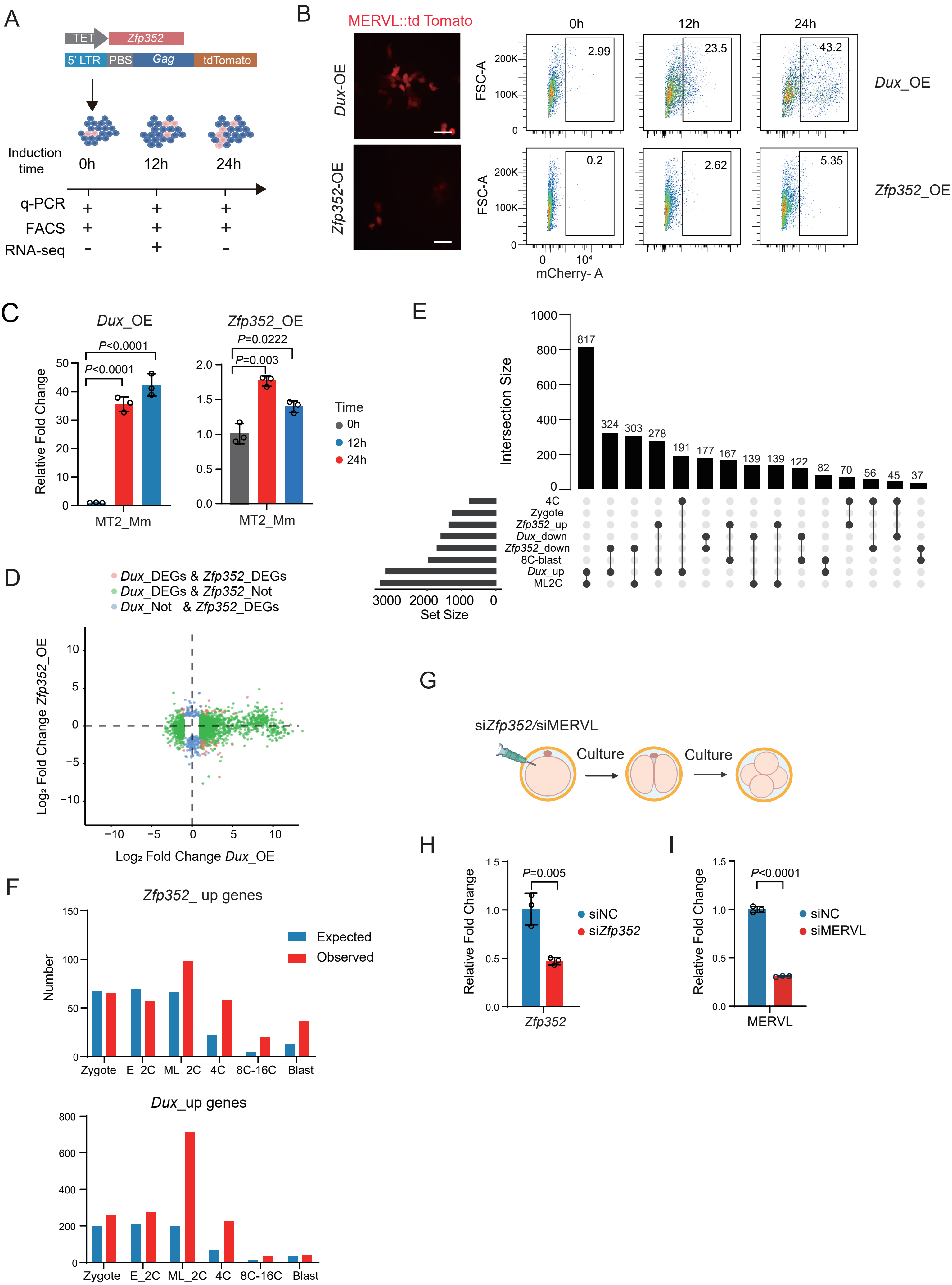

A
TET
Zfp352
5' LTR
PBS
Gag
tdTomato
Induction time
0h
12h
24h
q-PCR
FACS
RNA-seq
B
MERVL::td Tomato
Dux-OE
Zfp352-OE
0h
12h
24h
FSC-A
200K
100K
0
2.99
23.5
43.2
0.2
2.62
5.35
Dux_OE
Zfp352_OE
10⁴
mCherry- A
C
Dux_OE
Zfp352_OE
P<0.0001
P<0.0001
P=0.0222
P=0.003
Relative Fold Change
MT2_Mm
Time
0h
12h
24h
D
Dux_DEGs & Zfp352_DEGs
Dux_DEGs & Zfp352_Not
Dux_Not & Zfp352_DEGs
Log₂ Fold Change Zfp352_OE
Log₂ Fold Change Dux_OE
E
Intersection Size
817
324
303
278
191
177
167
139
139
122
82
70
56
45
37
4C
Zygote
Zfp352_up
Dux_down
Zfp352_down
8C-blast
Dux_up
ML2C
Set Size
F
Zfp352_ up genes
Expected
Observed
Number
Zygote
E_2C
ML_2C
4C
8C-16C
Blast
Dux_up genes
G
siZfp352/siMERVL
Culture
Culture
H
P=0.005
Relative Fold Change
siNC
siZfp352
Zfp352
I
P<0.0001
siNC
siMERVL
MERVL

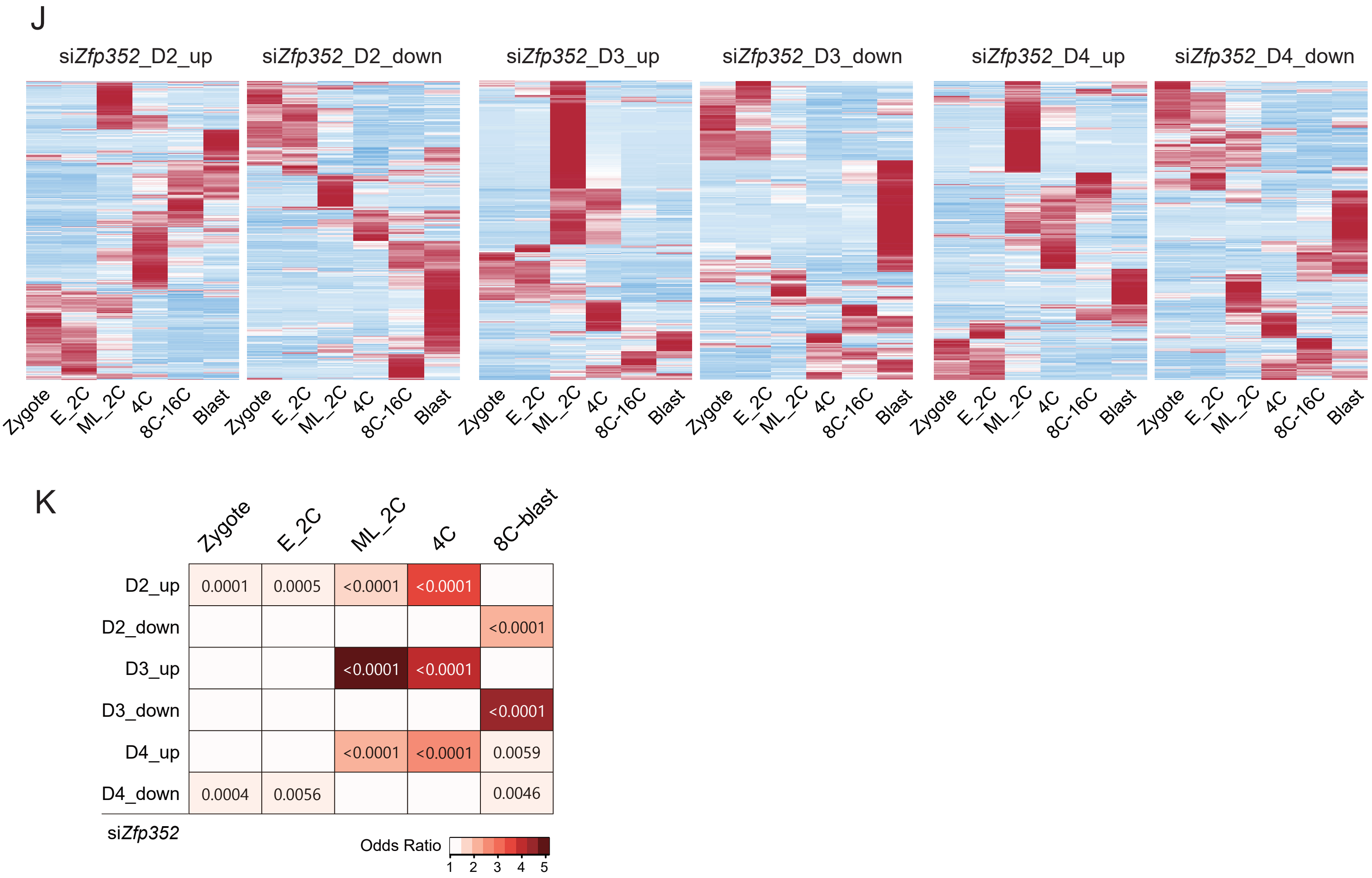
J
siZfp352_D2_up
siZfp352_D2_down
siZfp352_D3_up
siZfp352_D3_down
siZfp352_D4_up
siZfp352_D4_down
Zygote E_2C ML_2C 4C 8C-16C Blast
K
Zygote E_2C ML_2C 4C 8C-blast
D2_up 0.0001 0.0005 <0.0001 <0.0001
D2_down <0.0001
D3_up <0.0001 <0.0001
D3_down <0.0001
D4_up <0.0001 <0.0001 0.0059
D4_down 0.0004 0.0056 0.0046
siZfp352
Odds Ratio
1 2 3 4 5

Supplementary Figure 3: The transcriptome changes induced by ZFP352.

A) Schematics illustrating inducible *Zfp352* over-expression cell line constructed and the experimental design for transcriptome profiling.

B) The fluorescent pictures and FACS scatter plots showing the percentage of MERVL positive 2CLCs marked by MERVL::tdTomato reporter upon induction by *Dux* or *Zfp352* over-expression (scale bar = 75 μm).

C) The relative expression changes of MT2_Mm upon *Dux* or *Zfp352* over-expression for 12h and 24h.

D) Scatter plot showing the $\log_2$ gene expression fold change upon *Zfp352* or *Dux* over-expression. Genes up-regulated by both *Zfp352* or *Dux* over-expression were shown in red, genes up-regulated only upon *Zfp352* over-expression were shown in purple, and genes up-regulated only upon *Dux* over-expression were shown in green.

E) Bar plot showing the overlap of up-regulated and down-regulated genes (fold change [FC] > 1.5 / < -1.5 and Adjusted *P* value < 0.05) induced by *Zfp352* or *Dux* over-expression with embryo stage specific genes (GSE45719) (Two-sided fisher's exact test, multiple test by FDR method).

F) Barplots showing the observed and expected numbers of ZFP352 or DUX up-regulated genes in different embryonic stages (for Figure 2C).

G) Schematics illustrating the experimental design for *Zfp352* or MERVL siRNA injection into mouse zygotes in Figures 2E-F. Both embryo without injection or injected with scrambled siRNA were used as controls. Embryos were cultured to blastocyst stage to probe the developmental effect of siRNA.

H-I) RT-qPCR analysis showing the relative expression changes of *Zfp352* and MERVL at 2C stage upon *Zfp352* (H) or MERVL (I) siRNA injection; scrambled siRNA (siNC) was used as control respectively.

J-K) Differentially expressed genes from RNA-seq using day 2, 3, 4 mouse embryos upon *Zfp352* siRNA injection. (J) Z-score heatmaps for the developmental expression dynamics of up- or down-regulated DEGs upon si*Zfp352* injection**.** (K) enrichment analysis for the overlap between up- or down-regulated DEGs upon si*Zfp352* and the developmental stage marker genes in mouse embryo (one-sided fisher's exact test and multiple test by Benjamini & Hochberg method).

(C), (H-I) presented as mean ± SD, n = 3 biologically independent samples, two-sided unpaired t-test. (A) and (G) were created with BioRender.com.

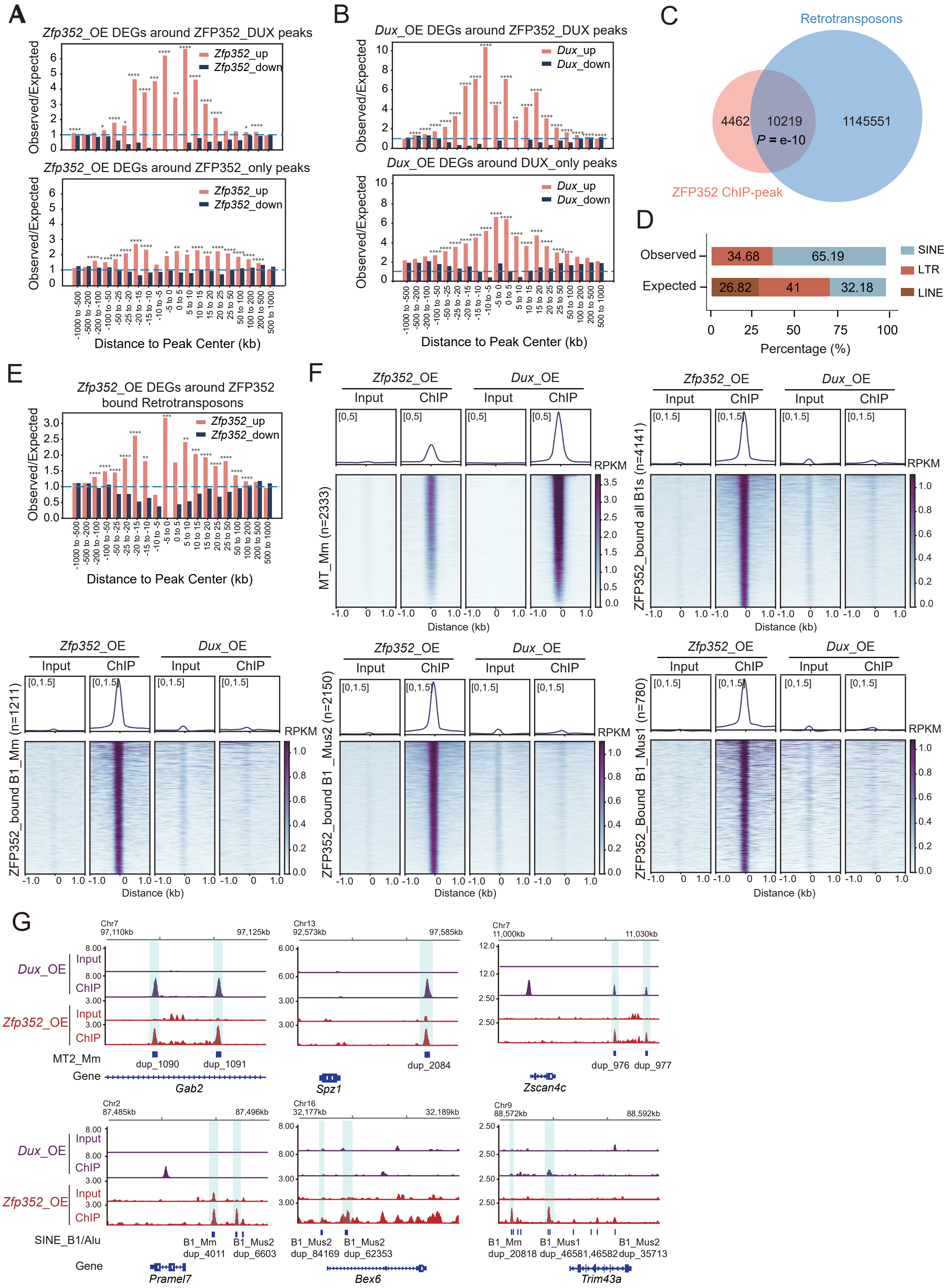
A
Zfp352_OE DEGs around ZFP352_DUX peaks
Zfp352_OE DEGs around ZFP352_only peaks
Distance to Peak Center (kb)
B
Dux_OE DEGs around ZFP352_DUX peaks
Dux_OE DEGs around DUX_only peaks
C
Retrotransposons
4462
10219
1145551
P = e-10
ZFP352 ChIP-peak
D
Observed
Expected
34.68
65.19
26.82
41
32.18
SINE
LTR
LINE
Percentage (%)
E
Zfp352_OE DEGs around ZFP352 bound Retrotransposons
F
MT_Mm (n=2333)
ZFP352_bound all B1s (n=4141)
ZFP352_bound B1_Mm (n=1211)
ZFP352_bound B1_Mus2 (n=2150)
ZFP352_Bound B1_Mus1 (n=780)
Distance (kb)
RPKM
G
Dux_OE
Zfp352_OE
MT2_Mm
SINE_B1/Alu
Gab2
Spz1
Zscan4c
Pramel7
Bex6
Trim43a

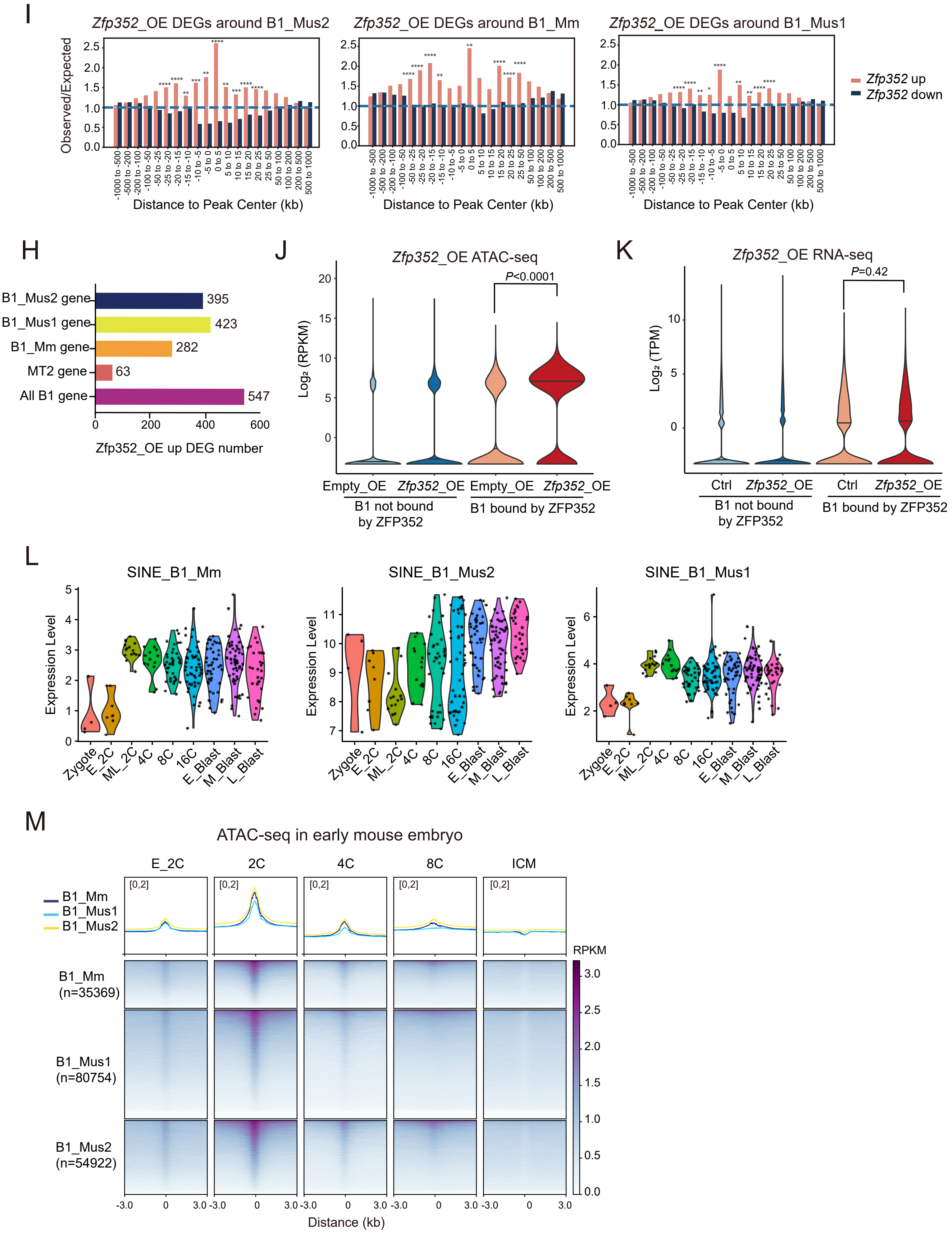
I
Zfp352_OE DEGs around B1_Mus2
Zfp352_OE DEGs around B1_Mm
Zfp352_OE DEGs around B1_Mus1
Observed/Expected
-1000 to -500
-500 to -200
-200 to -100
-100 to -50
-50 to -25
-25 to -20
-20 to -15
-15 to -10
-10 to -5
-5 to 0
0 to 5
5 to 10
10 to 15
15 to 20
20 to 25
25 to 50
50 to 100
100 to 200
200 to 500
500 to 1000
Distance to Peak Center (kb)
Zfp352 up
Zfp352 down
H
B1_Mus2 gene
B1_Mus1 gene
B1_Mm gene
MT2 gene
All B1 gene
395
423
282
63
547
Zfp352_OE up DEG number
J
Zfp352_OE ATAC-seq
P<0.0001
Log₂ (RPKM)
Empty_OE
Zfp352_OE
B1 not bound by ZFP352
B1 bound by ZFP352
K
Zfp352_OE RNA-seq
P=0.42
Log₂ (TPM)
Ctrl
Zfp352_OE
B1 not bound by ZFP352
B1 bound by ZFP352
L
SINE_B1_Mm
SINE_B1_Mus2
SINE_B1_Mus1
Expression Level
Zygote
E_2C
ML_2C
4C
8C
16C
E_Blast
M_Blast
L_Blast
M
ATAC-seq in early mouse embryo
E_2C
2C
4C
8C
ICM
[0,2]
B1_Mm
B1_Mus1
B1_Mus2
RPKM
B1_Mm (n=35369)
B1_Mus1 (n=80754)
B1_Mus2 (n=54922)
-3.0
0
3.0
Distance (kb)

Supplementary Figure 4: ZFP352 binding was enriched on MT2_Mm and SINE_B1/Alu

A) The barplot showing the association between *Zfp352* over-expression DEGs and different types of ChIP-seq peaks as analyzed by RAD. ZFP352_DUX peaks represent DUX and ZFP352 co-bound peaks, ZFP352_only peaks represent ZFP352 peaks not bound by DUX.
B) The barplot showing the association between *Dux* over-expression DEGs and different types of ChIP-seq peaks as analyzed by RAD. ZFP352_DUX peaks represent DUX and ZFP352 co-bound peaks, DUX_only peaks represent DUX peaks not bound by ZFP352.
C) Venn diagram showing the overlap between ZFP352 peaks and retrotransposons. *P* value = e-10 (close to 0, using bedtools one-sided Fisher test function).
D) The barplot showing the percentage of different retrotransposon classes bound by ZFP352. 'Expected' represents the genomic average assuming random distribution.
E) Barplots showing the association between ZFP352 peaks which were also overlapping with retrotransposons and *Zfp352* over-expression induced DEGs as analyzed by RAD.
F) Metaplots and heatmaps of the ZFP352 and DUX ChIP-seq signal over different retrotransposon sub-families, including MT2_Mm, ZFP352-bound B1_Mus2, ZFP352-bound B1_Mm, ZFP352-bound B1_Mus1, and ZFP352-bound all three combined SINE_B1s sub-families.
G) Coverage plots of DUX (purple) and ZFP352 (red) ChIP-seq signals over selected MT2_Mm and nearby genes (*Gab2*, *Spz1, Zscan4c*), as well as SINE_B1/Alu and nearby genes (*Pramel7, Bex6, Trim43a*).
H) Barplot showing the association between *Zfp352* over-expression DEGs and different types of SINE_B1/Alu sub-families bound by ZFP352.
I) Bar graph showing the gene numbers for different sub-families of ZFP352_B1 genes and ZFP352_MT2 genes.
J-K) Violin plot showing the ATAC-seq signal (J) or RNA expression level (K) differences for ZFP352 bound SINE_B1s or ZFP352 unbound SINE_B1s in mESCs without or with *Zfp352* over-expression.
L) Violin plot showing the expression dynamic of different sub-families of SINE_B1 at different mouse embryo developmental stages. The data were analysed using single cell RNA-seq data of pre-implantation mouse embryos (GSE45719).
M) Metaplots and heapmaps of ATAC-seq signals for different SINE_B1 sub-families at different mouse embryo developmental stages (GSE66390).
(A), (B), (E) and (H) presented as two-sided hypergeometric test; in (J) and (K): two-sided Wilcoxon test.

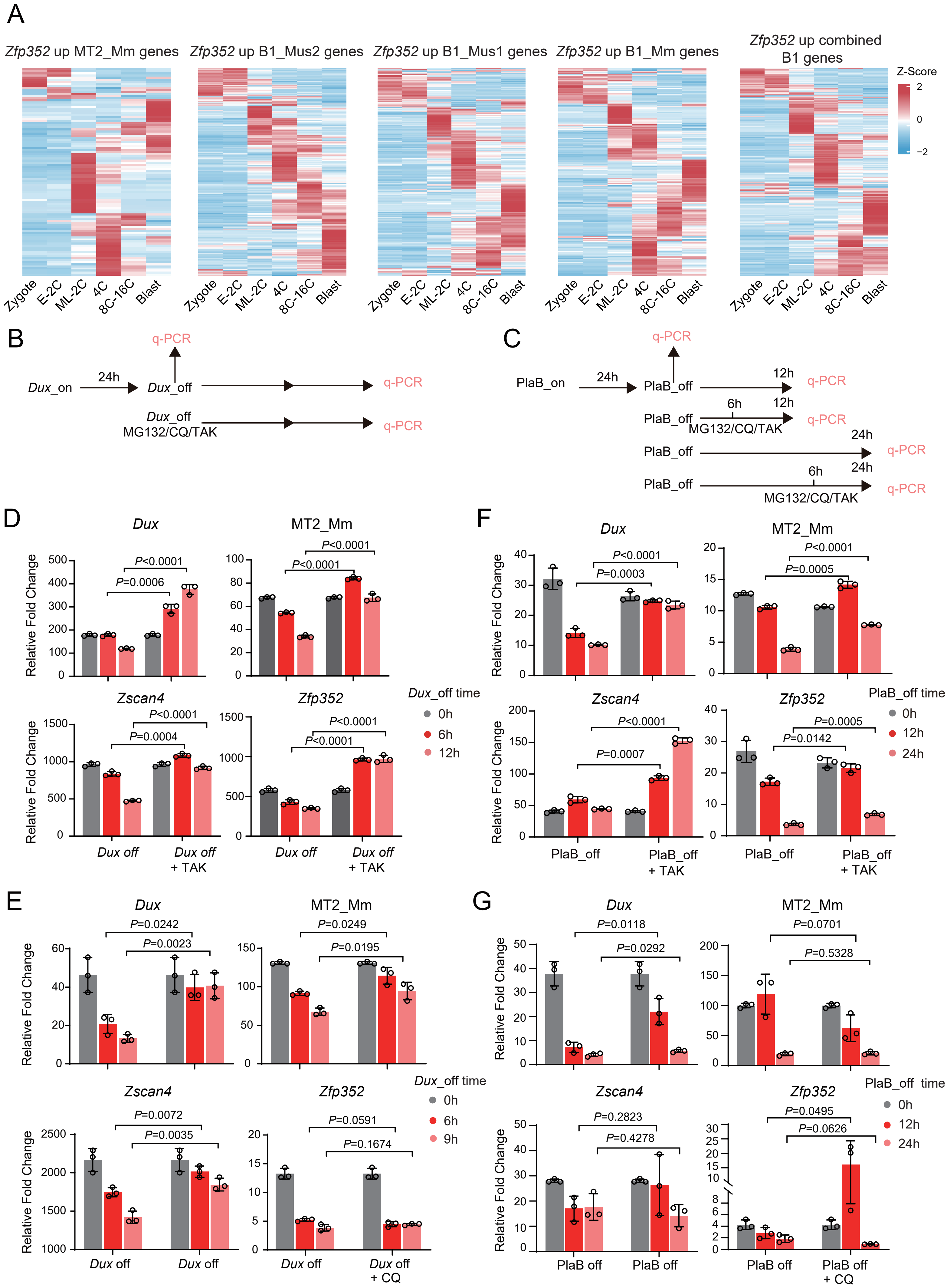

A
Zfp352 up MT2_Mm genes
Zfp352 up B1_Mus2 genes
Zfp352 up B1_Mus1 genes
Zfp352 up B1_Mm genes
Zfp352 up combined B1 genes
Z-Score
2
0
−2
Zygote
E-2C
ML-2C
4C
8C-16C
Blast
B
Dux_on
24h
Dux_off
q-PCR
Dux_off MG132/CQ/TAK
C
PlaB_on
24h
PlaB_off
6h
12h
24h
PlaB_off MG132/CQ/TAK
q-PCR
D
Dux
MT2_Mm
Zscan4
Zfp352
Relative Fold Change
P<0.0001
P=0.0006
P=0.0004
Dux off
Dux off + TAK
Dux_off time
0h
6h
12h
F
P=0.0003
P=0.0005
P=0.0007
P=0.0142
P=0.0005
PlaB_off
PlaB_off + TAK
PlaB_off time
0h
12h
24h
E
P=0.0242
P=0.0023
P=0.0249
P=0.0195
P=0.0072
P=0.0035
P=0.0591
P=0.1674
Dux off
Dux off + CQ
Dux_off time
0h
6h
9h
G
P=0.0118
P=0.0292
P=0.0701
P=0.5328
P=0.2823
P=0.4278
P=0.0495
P=0.0626
PlaB off
PlaB off + CQ
PlaB_off time
0h
12h
24h

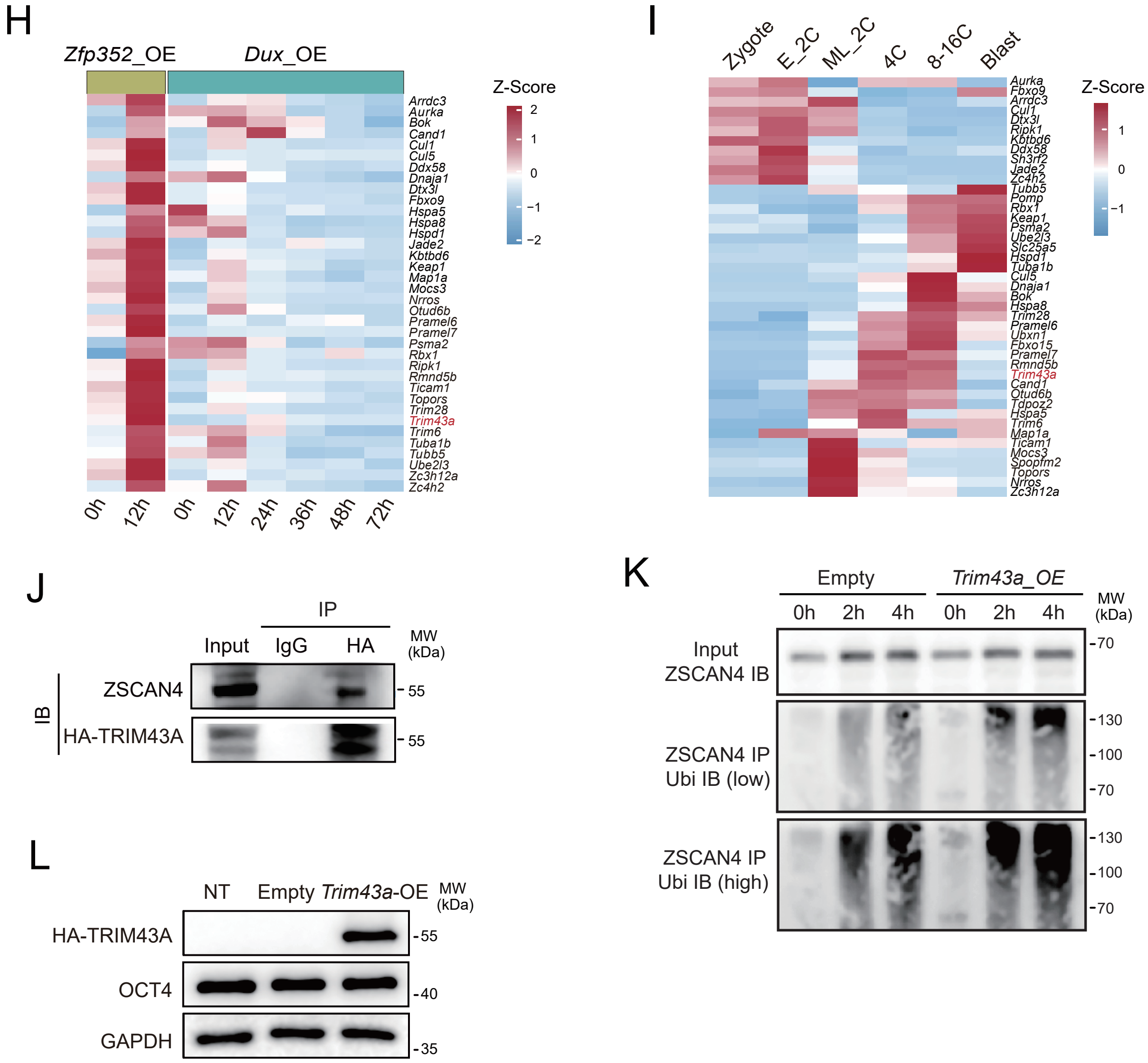

H
Zfp352_OE
Dux_OE
Z-Score
2
1
0
−1
−2
Arrdc3
Aurka
Bok
Cand1
Cul1
Cul5
Ddx58
Dnaja1
Dtx3l
Fbxo9
Hspa5
Hspa8
Hspd1
Jade2
Kbtbd6
Keap1
Map1a
Mocs3
Nrros
Otud6b
Pramel6
Pramel7
Psma2
Rbx1
Ripk1
Rmnd5b
Ticam1
Topors
Trim28
Trim43a
Trim6
Tuba1b
Tubb5
Ube2l3
Zc3h12a
Zc4h2
0h
12h
0h
12h
24h
36h
48h
72h
I
Zygote
E_2C
ML_2C
4C
8-16C
Blast
Z-Score
1
0
−1
Aurka
Fbxo9
Arrdc3
Cul1
Dtx3l
Ripk1
Kbtbd6
Ddx58
Sh3rf2
Jade2
Zc4h2
Tubb5
Pomp
Rbx1
Keap1
Psma2
Ube2l3
Slc25a5
Hspd1
Tuba1b
Cul5
Dnaja1
Bok
Hspa8
Trim28
Pramel6
Ubxn1
Fbxo15
Pramel7
Rmnd5b
Trim43a
Cand1
Otud6b
Tdpoz2
Hspa5
Trim6
Map1a
Ticam1
Mocs3
Spopfm2
Topors
Nrros
Zc3h12a
J
IP
Input
IgG
HA
MW (kDa)
IB
ZSCAN4
HA-TRIM43A
55
55
K
Empty
Trim43a_OE
0h
2h
4h
0h
2h
4h
MW (kDa)
Input ZSCAN4 IB
70
ZSCAN4 IP Ubi IB (low)
130
100
70
ZSCAN4 IP Ubi IB (high)
130
100
70
L
NT
Empty
Trim43a-OE
MW (kDa)
HA-TRIM43A
55
OCT4
40
GAPDH
35

Supplementary Figure 5: protein degradation contributes to 2CL state dissolution.

A) Heatmaps showing the expression dynamics of ZFP352 up-regulated genes with ZFP352 bound MT2_Mm peaks (ZFP352 up MT2_Mm genes), or ZFP352 up-regulated genes with different ZFP352 bound SINE_B1 peaks (ZFP352 up B1_Mus2 genes, ZFP352 up B1_Mus1 genes, ZFP352 up B1_Mm genes, ZFP352 up all B1 genes) at different mouse embryo stages. Color code represents the Z-score of expression changes.

B-C) Schematics illustrating the experimental design for Figure 4C, S4D and S4E (B). and Figures 4D, S4F and S4G (C) respectively.

D) Barplots showing the expression changes of key 2C genes upon TAK243 treatment during the exit from DUX induced 2CL state. Genes expression changes at 0h, 6h and 12h upon releasing from 2CL state were measured by RT-qPCR.

E) Barplots showing the expression changes of key 2C genes upon CQ treatment during the exit from DUX induced 2CL state. Genes expression changes at 0h, 6h and 9h upon releasing from 2CL state were measured by RT-qPCR.

F-G) Barplots showing the expression changes of key 2C genes upon TAK243 treatment (F) or CQ treatment (G) during the exit from PlaB induced TBLC state. Genes expression changes at 0h, 12h and 24h upon releasing from TBLC state were measured by RT-qPCR.

H) Heatmap showing the expression dynamics of ZFP352 up-regulated SINE_B1/Alu nearby genes, which fall into the ubiquitin and proteasome-related GO terms, upon *Zfp352* over-expression for 12h and *Dux* over-expression for 12h, 24h, 36h, 48h, 72h.

I) Heatmap showing the expression dynamics of ZFP352 up-regulated SINE_B1/Alu nearby genes, which fall into the ubiquitin and proteasome-related GO terms, at different mouse embryonic stages.

J) Co-immunoprecipitation of TRIM43A and ZSCAN4 in HEK293T cells over-expressing HA-*Trim43a* and ZSCAN4. HA-TRIM43A was pulled down to detect ZSCAN4.

K) Western blot showing the ZSCAN4 ubiquitination by TRIM43A. *Zscan4* was over-expressed in HEK293T cells with or without *Trim43a* over-expression. ZSCAN4 was pulled down and ubiquitination level was detected.

L) Western blot showing the effect of *Trim43a* over-expression on OCT4 protein level in mESCs.

(D-G) presented as mean ± SD, n = 3 biologically independent samples, two-sided unpaired t-test.

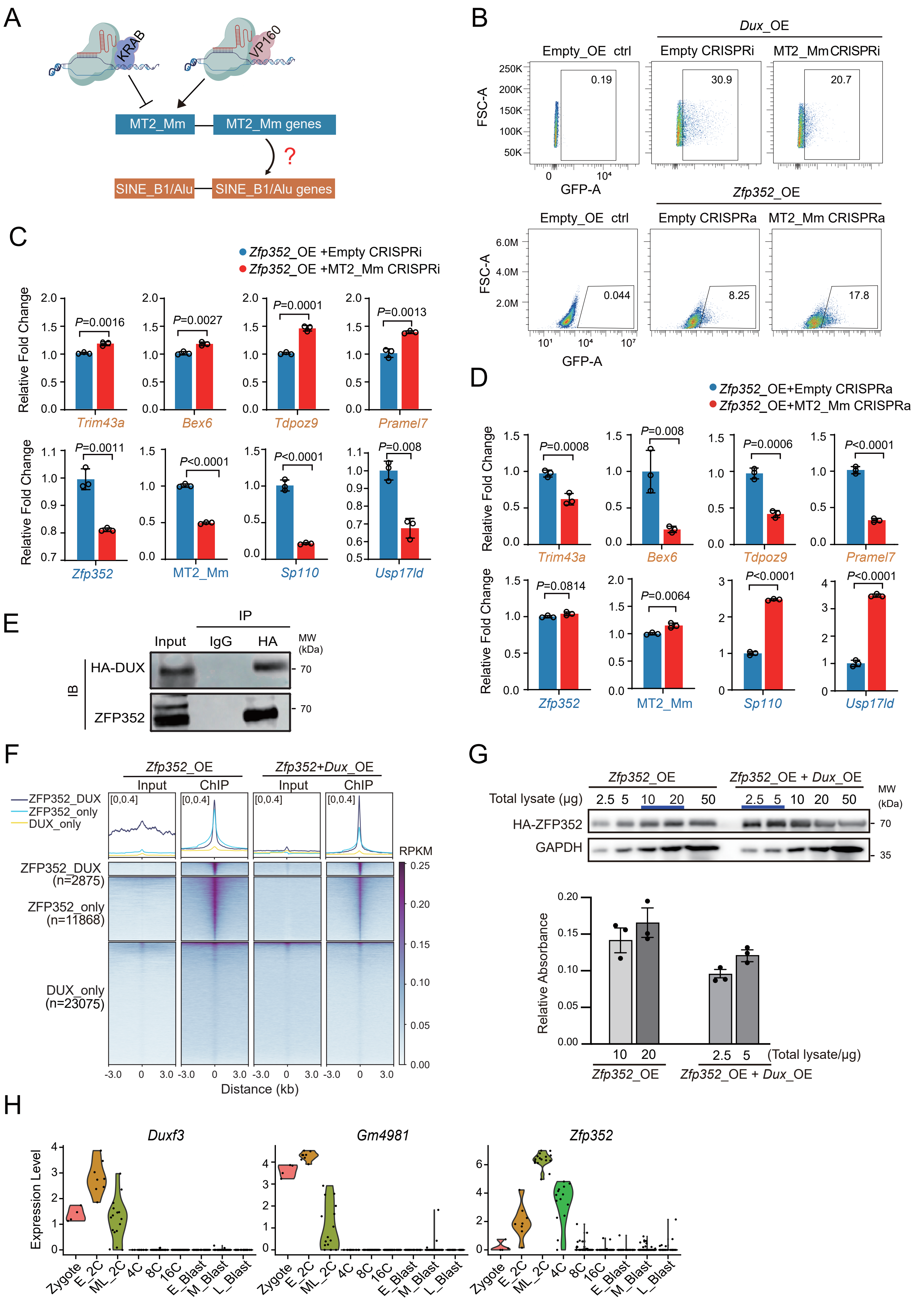

A
KRAB
VP160
MT2_Mm
MT2_Mm genes
?
SINE_B1/Alu
SINE_B1/Alu genes
B
Dux_OE
Empty_OE ctrl
Empty CRISPRi
MT2_Mm CRISPRi
0.19
30.9
20.7
FSC-A
GFP-A
Zfp352_OE
Empty_OE ctrl
Empty CRISPRa
MT2_Mm CRISPRa
0.044
8.25
17.8
C
Zfp352_OE +Empty CRISPRi
Zfp352_OE +MT2_Mm CRISPRi
Relative Fold Change
P=0.0016
P=0.0027
P=0.0001
P=0.0013
Trim43a
Bex6
Tdpoz9
Pramel7
P=0.0011
P<0.0001
P<0.0001
P=0.008
Zfp352
MT2_Mm
Sp110
Usp17ld
D
Zfp352_OE+Empty CRISPRa
Zfp352_OE+MT2_Mm CRISPRa
P=0.0008
P=0.008
P=0.0006
P<0.0001
P=0.0814
P=0.0064
P<0.0001
P<0.0001
E
IP
Input
IgG
HA
MW (kDa)
IB
HA-DUX
ZFP352
70
F
Zfp352_OE
Zfp352+Dux_OE
Input
ChIP
ZFP352_DUX
ZFP352_only
DUX_only
ZFP352_DUX (n=2875)
ZFP352_only (n=11868)
DUX_only (n=23075)
RPKM
Distance (kb)
G
Zfp352_OE
Zfp352_OE + Dux_OE
Total lysate (μg)
HA-ZFP352
GAPDH
Relative Absorbance
(Total lysate/μg)
H
Duxf3
Gm4981
Zfp352
Expression Level
Zygote
E_2C
ML_2C
4C
8C
16C
E_Blast
M_Blast
L_Blast

I

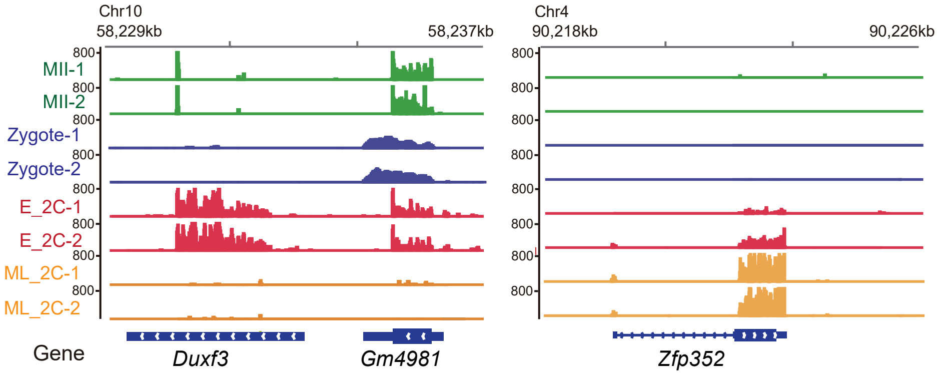

Chr10
58,229kb
58,237kb
Chr4
90,218kb
90,226kb
800
MII-1
MII-2
Zygote-1
Zygote-2
E_2C-1
E_2C-2
ML_2C-1
ML_2C-2
Gene
Duxf3
Gm4981
Zfp352

J

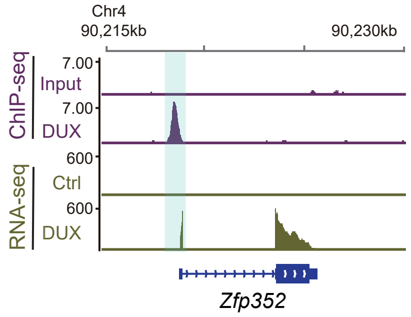

Chr4
90,215kb
90,230kb
ChIP-seq
7.00
Input
DUX
RNA-seq
600
Ctrl
DUX
Zfp352

K

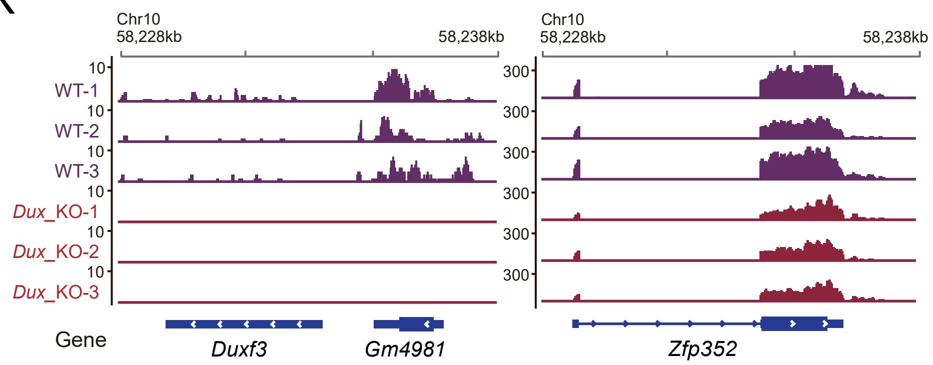

Chr10
58,228kb
58,238kb
10
300
WT-1
WT-2
WT-3
*Dux*_KO-1
*Dux*_KO-2
*Dux*_KO-3
Gene
Duxf3
Gm4981
Zfp352

Supplementary Figure 6: DUX modulates ZFP352 binding affinity.

A) Schematics illustrating the experimental design for CRIPSR/Cas9-mediated activation or inactivation of MT2_Mm towards the expression of SINE_B1/Alu nearby genes. Created with BioRender.com.

B) FACS analysis of MERVL positive cells shown by MERVL::GFP reporter upon CRISPRi targeting MT2_Mm in *Dux*_OE cells or CRISPRa targeting MT2_Mm in *Zfp352*_OE cells. Empty CRISPRi or empty CRISPRa without targeting effect was used as control.

C-D) The relative fold change of *Zfp352*, MT2_Mm, nearby 2C genes (*Sp110, Usp17ld*) and SINE_B1/Alu nearby genes (*Trim43a*, *Bex6*, *Tdpoz9*, *Pramel7*) upon over-expression of CRISPRi (C) or CRISPRa (D) targeting MT2_Mm in *Zfp352* over-expressed mESCs. The expression was normalized to mESCs over-expressing non-targeting CRISPRi or CRISPRa.

E) Co-immunoprecipitation of ZFP352 and DUX in HEK293T cell over-expressing *Zfp352* and HA-*Dux*. HA-DUX was pulled down to detect ZFP352.

F) Metaplots and heatmaps of ZFP352 ChIP-seq signal over ZFP352_DUX overlapping peak center (purple), ZFP352_only peak center (blue) and DUX_only peak center (yellow) as defined in Figure 3A. ZFP352 ChIP-seq were perform in *Zfp352* over-expressing or *Zfp352_Dux* co-expressing mESCs respectively.

G) ELISA showing the binding of ZFP352 with SINE_B1 DNA probe. Total cell lysates from *Zfp352* or *Zfp352_Dux* over-expressed in HEK293T cells were loaded at different protein amount. The upper western blot showing ZFP352 protein level with different lysate amount. The one with similar ZFP352 levels in *Zfp352*_OE and *Zfp352*_OE+*Dux*_OE conditions (underlined) was loaded for ELISA to detect ZFP352 binding onto SINE_B1 (lower bar chart).

H) Violin plot showing the expression dynamic of *Dux* cluster genes and *Zfp352* at different embryo developmental stages. The data were from single cell RNA-seq of pre-implantation mouse embryos (GSE45719).

I) Coverage plot of Ribo-seq signal at early embryonic stages (MII, Zygote, Early 2C and Mid_late 2C) over *Dux* cluster genes and *Zfp352* loci (GSE165782).

J) Coverage plot of DUX ChIP-seq (purple) and RNA-seq (green) signals over *Zfp352* loci in *Dux* over-expressing mESCs.

K) Coverage plot of RNA-seq signal over *Dux* cluster genes and *Zfp352* loci in wide type mouse embryos (purple) and *Dux* cluster gene knockout mouse embryos (red) (GSE121746).

(C-D) and (G) presented as mean ± SD, n = 3 biologically independent samples, two-sided unpaired t-test.

Figure 1D

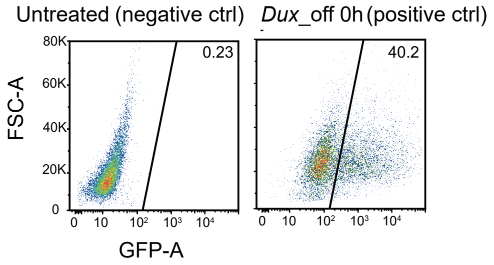

Figure 1F

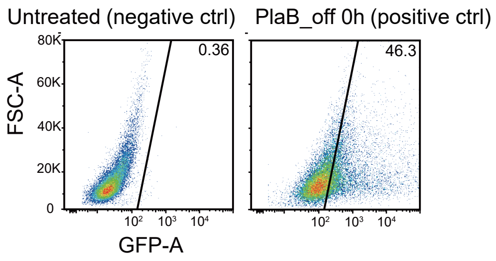

Supplementary Figure 3B

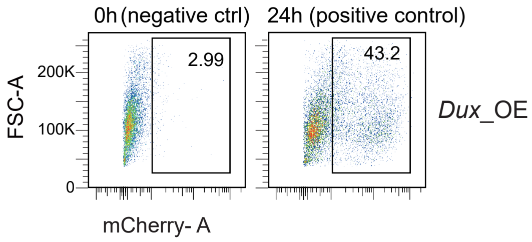

Supplementary Figure 6B

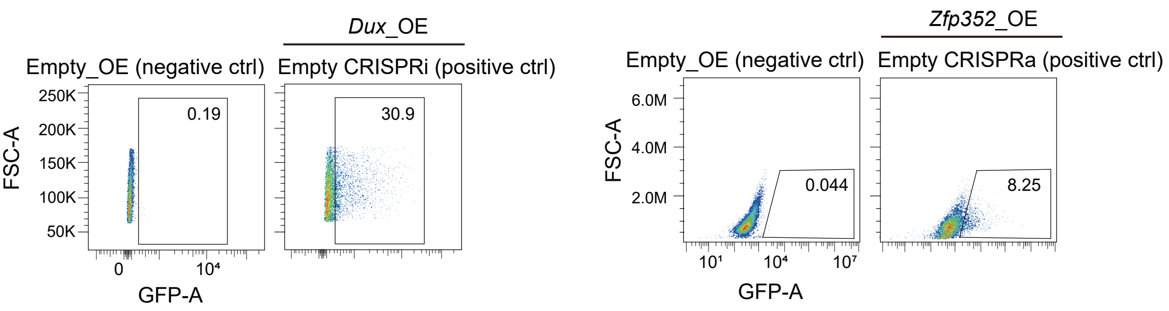

Supplementary Figure 7: Gating strategy for FACS data in the figures.
For Figure 1D, the untreated sample was used as negative control, *Dux*_off 0h was used as positive control. For Figure 1F, the untreated sample was used as negative control, PlaB_off 0h was used as positive control. For Supp Figure 2B, the 0h sample was used as negative control, 24h sample was used as positive control. For Supp Figure 5B, the empty_OE sample was used as negative control, empty CRISPRi or CRISPRa sample was used as positive control. In all these experiments, the gates were set to make the differences in the percentage of GFP+ve or mCherry+ve subpopulations between the positive and negative controls stay the maximum.

Figure 4E

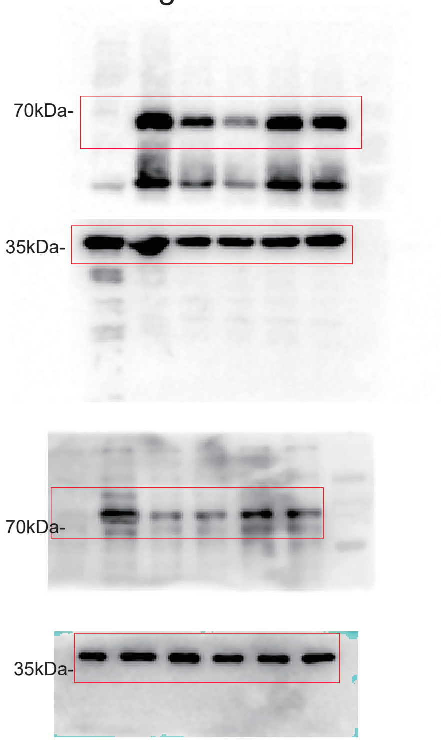

Figure 4H

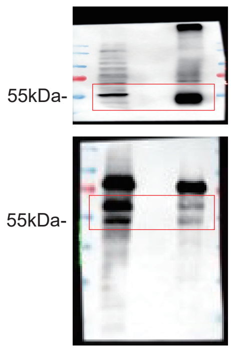

Figure 4I

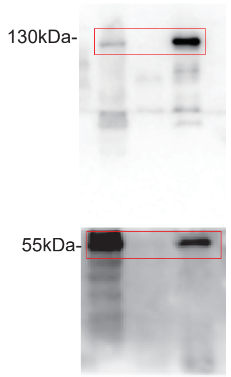

Figure 4K

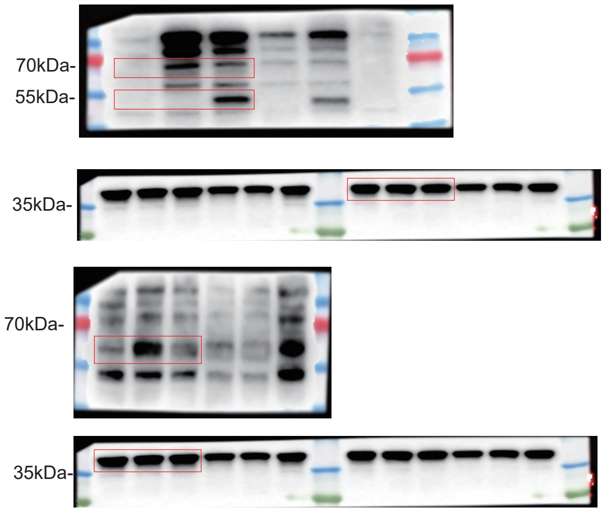

Figure 4J

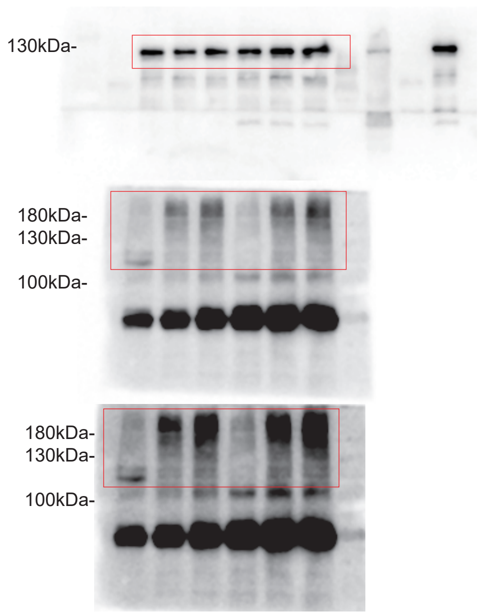

Figure 5E

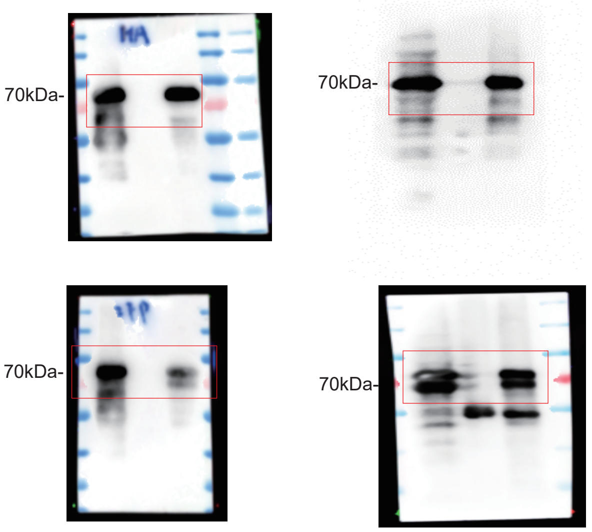

Supplementary Figure 8: Uncropped scan of gels for main figures.

Supplementary Fig.2B

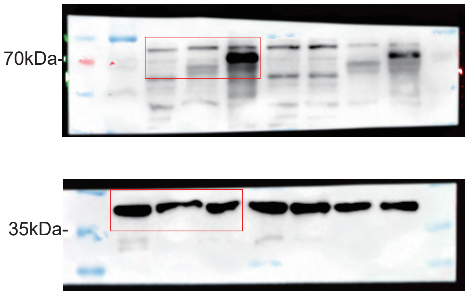

Supplementary Fig.2E

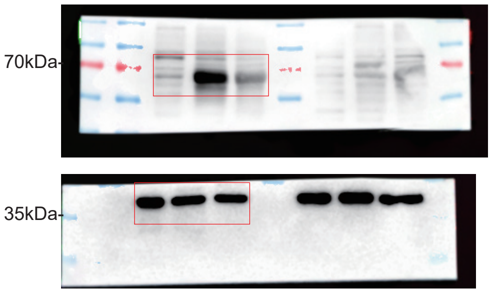

Supplementary Fig.2H

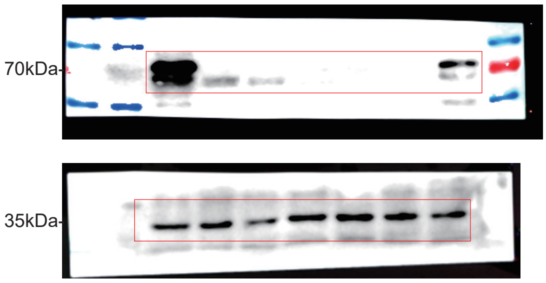

Supplementary Fig.5J

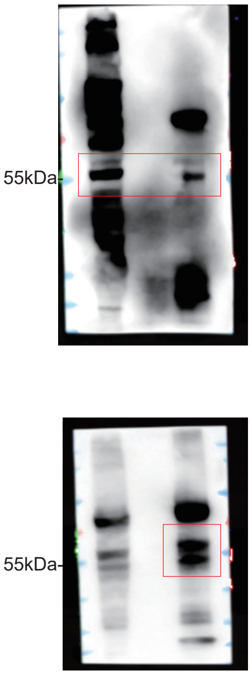

Supplementary Fig.5K

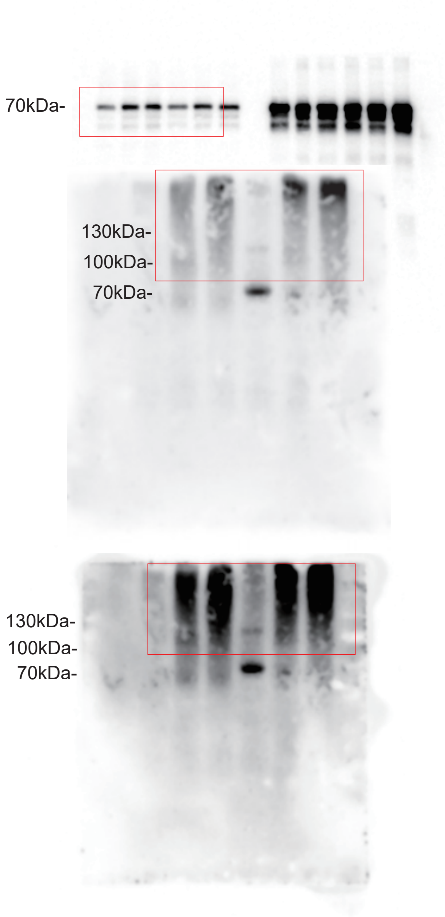

Supplementary Fig.5L

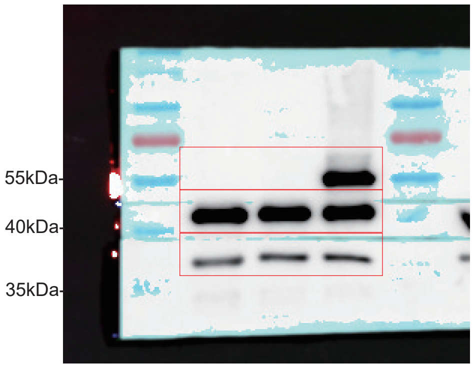

Supplementary Fig.6E

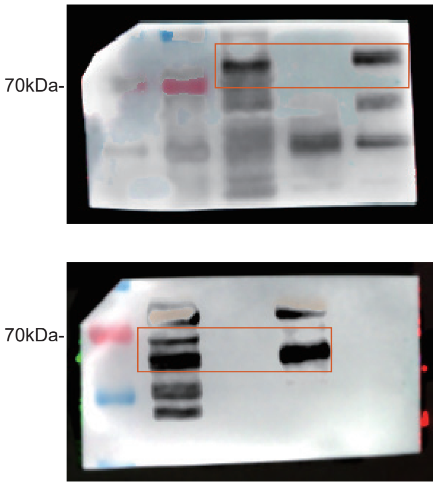

Supplementary Fig.6G

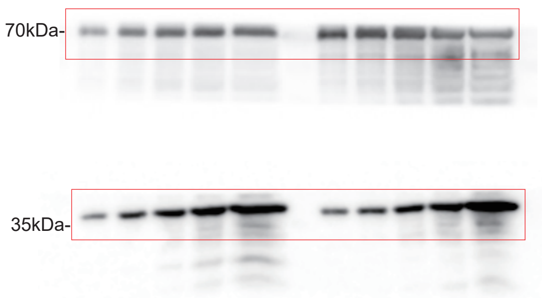

Supplementary Figure 9: Uncropped scan of gels for supplementary figures.
