# Supplementary material for: Selective binding of retrotransposons by ZFP352 facilitates the timely dissolution of totipotency network
Source: Nat Commun. 2023 Jun 20;14:3646. doi: 10.1038/s41467-023-39344-1 (PMC10281998; doi:10.1038/s41467-023-39344-1)
Supplement: Supplementary file 2 — Description of Additional Supplementary Files [file 41467_2023_39344_MOESM2_ESM.pdf]

Description of Additional Supplementary Files

File Name: Supplementary Data 1
Description: Gene list for different DEG clusters of *Dux* over-expressing mESCs and for DEG clusters of mouse early embryo development.
Gene list of DEG clusters based on the temporal expression pattern upon *Dux* over-expression for different time points, and gene list of DEG clusters based on the mouse early embryo development stages. These two gene lists were shown in separate sheets. Related to Figure.1 and Supplementary Figure.1.

File Name: Supplementary Data 2
Description: List of DEGs and DETEs in *Dux* over-expressing mESCs.
List of DEGs (fold change > 1.5 or < -1.5, Adjusted *P* values < 0.05) and DETEs (fold change > 1.5 or < -1.5, Adjusted *P* values < 0.05) between mESCs with *Dux* induction for 12h, 24h, 36h, 48h, 72h on and mESCs before *Dux* induction (two-sided fisher' exact test; multiple test by FDR method). DEGs and DETEs were shown in separate sheets. Related to Figure.1 and 2, Supplementary Figure.1, 2 and 3.

File Name: Supplementary Data 3
Description: List of DEGs and DETEs in *Zfp352* over-expressing mESCs.
List of DEGs (fold change > 1.5 or < -1.5, Adjusted *P* values < 0.05) and DETEs (fold change > 1.5 or < -1.5, Adjusted *P* values < 0.05) between mESCs over-expressing *Zfp352* for 12h and mESCs not over-expressing *Zfp352* (two-sided fisher' exact test; multiple test by FDR method). DEGs and DETEs were shown in separate sheets. Related to Figure.2-4, Supplementary Figure.3-5.

File Name: Supplementary Data 4
Description: The enrichment scores of the top 50 enriched hexamers in the sequences of ZFP352_bound SINE_B1/Alu and MT2_Mm.
List of top 50 enriched hexamers and their enrichment scores of ZFP352_bound SINE_B1/Alu and MT2_Mm. The enrichment scores correspond to the proportion of a hexamer among all hexamers in ZFP352_bound SINE_B1/Alu and MT2_Mm sequences. Related to Figure.5A.

File Name: Supplementary Data 5
Description: List for the oligonucleotide sequences.
