# Supplementary material for: Selective binding of retrotransposons by ZFP352 facilitates the timely dissolution of totipotency network
Source: Nat Commun. 2023 Jun 20;14:3646. doi: 10.1038/s41467-023-39344-1 (PMC10281998; doi:10.1038/s41467-023-39344-1)
Supplement: Supplementary file 8 — Reporting Summary [file 41467_2023_39344_MOESM8_ESM.pdf]

nature portfolio

Corresponding author(s): Dan Zhang, Yang Shen, Hongqing Liang

Last updated by author(s): 25th May 2023

Reporting Summary

Nature Portfolio wishes to improve the reproducibility of the work that we publish. This form provides structure for consistency and transparency in reporting. For further information on Nature Portfolio policies, see our Editorial Policies and the Editorial Policy Checklist.

Statistics

For all statistical analyses, confirm that the following items are present in the figure legend, table legend, main text, or Methods section.

n/a	Confirmed	
☐	☒	The exact sample size (*n*) for each experimental group/condition, given as a discrete number and unit of measurement
☐	☒	A statement on whether measurements were taken from distinct samples or whether the same sample was measured repeatedly
☐	☒	The statistical test(s) used AND whether they are one- or two-sided<br>*Only common tests should be described solely by name; describe more complex techniques in the Methods section.*
☒	☐	A description of all covariates tested
☐	☒	A description of any assumptions or corrections, such as tests of normality and adjustment for multiple comparisons
☐	☒	A full description of the statistical parameters including central tendency (e.g. means) or other basic estimates (e.g. regression coefficient) AND variation (e.g. standard deviation) or associated estimates of uncertainty (e.g. confidence intervals)
☐	☒	For null hypothesis testing, the test statistic (e.g. *F*, *t*, *r*) with confidence intervals, effect sizes, degrees of freedom and *P* value noted<br>*Give P values as exact values whenever suitable.*
☒	☐	For Bayesian analysis, information on the choice of priors and Markov chain Monte Carlo settings
☒	☐	For hierarchical and complex designs, identification of the appropriate level for tests and full reporting of outcomes
☐	☒	Estimates of effect sizes (e.g. Cohen's *d*, Pearson's *r*), indicating how they were calculated

Our web collection on statistics for biologists contains articles on many of the points above.

Software and code

Policy information about availability of computer code

Data collection	MERVL+ve cells were analysed by BD Fortessa machine<br>qPCR was performed using Roche LC480II machine.<br>Western blot images were collected by Azure-600 system.<br>Luciferase activity was measured with the dual luciferase assay system (Promega, E1910)
Data analysis	Software used: STAR(v2.7.0e), FeatureCounts (v2.0.0), Seurat (v3.0.0), FastQC (v0.11.8), cutadapt (v2.9),e dgeR (v3.30.3), clusterProfiler (v3.12.0), GeneOverlap (v1.30.0), nf-core/chipseq" (v1.1.0), TrimGalore (v0.5.0), BWA (v0.7.17),PICARD (v2.19.0), SAMtools (v1.9), MACS2 (v2.1.2), Deeptools (v3.4.3), Homer (v4.7), BEDtools (v2.29.2), Jellyfish (v2.3.0), pheatmap (v1.0.12), ComplexHeatmap(v2.0.0), Flowjo X V10, Synergy2 plate reader (BioTek)<br>The code is available at GitHub (https://github.com/zhengyilii/ZFP352_2C/tree/Code_Data).

For manuscripts utilizing custom algorithms or software that are central to the research but not yet described in published literature, software must be made available to editors and reviewers. We strongly encourage code deposition in a community repository (e.g. GitHub). See the Nature Portfolio guidelines for submitting code & software for further information.

Data

Policy information about availability of data

All manuscripts must include a data availability statement. This statement should provide the following information, where applicable:

- Accession codes, unique identifiers, or web links for publicly available datasets
- A description of any restrictions on data availability
- For clinical datasets or third party data, please ensure that the statement adheres to our policy

The RNA-seq, ATAC-seq, ChIP-seq and embryo RNA-seq data generated in this study have been deposited at GEO database under the accession code GSE222636 (https://www.ncbi.nlm.nih.gov/geo/query/acc.cgi?&acc=GSE222636) and are publicly available as of the date of publication. The raw data for the bar plot and micrographs generated in this study are provided in the source data file. Primers used in the manuscript is available in Supplementary Data 5.
Data referenced in this study are available in Gene Expression Omnibus (GEO) with the references and accession numbers:
DUX CHIP data (GEO: GSE95517)
https://www.ncbi.nlm.nih.gov/geo/query/acc.cgi?acc=GSE95517
Mouse embryo scRNA-seq (GEO: GSE45719)
https://www.ncbi.nlm.nih.gov/geo/query/acc.cgi?acc=GSE45719
Dux_OE 2C-enrty scRNA-seq (GEO: GSE121459)
https://www.ncbi.nlm.nih.gov/geo/query/acc.cgi?acc=GSE121459
Dux_OE 2C-exit scRNA-seq (GEO: GSE133234)
https://www.ncbi.nlm.nih.gov/geo/query/acc.cgi?acc=GSE133234
Mouse embryo ATAC-seq (GEO: GSE66390)
https://www.ncbi.nlm.nih.gov/geo/query/acc.cgi?acc=GSE66390
Mouse embryo Ribo-seq (GEO: GSE165782)
https://www.ncbi.nlm.nih.gov/geo/query/acc.cgi?acc=GSE165782
Mouse embryo Dux_KO RNA-seq (GEO: GSE121746)
https://www.ncbi.nlm.nih.gov/geo/query/acc.cgi?acc=GSE121746

Human research participants

Policy information about studies involving human research participants and Sex and Gender in Research.

Reporting on sex and gender	N/A
Population characteristics	N/A
Recruitment	N/A
Ethics oversight	N/A

Note that full information on the approval of the study protocol must also be provided in the manuscript.

Field-specific reporting

Please select the one below that is the best fit for your research. If you are not sure, read the appropriate sections before making your selection.

☒ Life sciences ☐ Behavioural & social sciences ☐ Ecological, evolutionary & environmental sciences

For a reference copy of the document with all sections, see nature.com/documents/nr-reporting-summary-flat.pdf

Life sciences study design

All studies must disclose on these points even when the disclosure is negative.

Sample size	No statistical methods were used to predetermine the sample sized. Three or more sample sizes were chosen to provide enough replicates for statistical analysis, and the exact sample size of each experiment was determined by availability of biological samples. Sample size and statistical analysis are provided in figure legends and method session.
Data exclusions	No data were excluded from the analysis.
Replication	The experiments were independently replicated at least twice and repeated at least three times within each of the experimental runs.
Randomization	The embryos experiments were randomized by pooling all the fertilized eggs into one culture medium droplet, and then distributing by mouse-pipetting the equal number of embryos into different culture medium droplets for different subsequent treatments. Cell experiments contain transfection, transduction, luciferase expression assay, Co-immunoprecipitation, Western blotting, Flow cytometry, CRISPRi/CRISPRa and ELISA assay. These cells experiments were all done by distributing cells into culture wells and treated with different

conditions in repeats.

Blinding	Blinding was performed in embryo experiments. For all the cell biological experiments, investigators were not blinded to group allocation for data collection and analysis since the same investigator designed and performed the experiments.

Reporting for specific materials, systems and methods

We require information from authors about some types of materials, experimental systems and methods used in many studies. Here, indicate whether each material, system or method listed is relevant to your study. If you are not sure if a list item applies to your research, read the appropriate section before selecting a response.

Materials & experimental systems

n/a	Involved in the study
☐	☒ Antibodies
☐	☒ Eukaryotic cell lines
☒	☐ Palaeontology and archaeology
☐	☒ Animals and other organisms
☒	☐ Clinical data
☒	☐ Dual use research of concern

Methods

n/a	Involved in the study
☐	☒ ChIP-seq
☐	☒ Flow cytometry
☒	☐ MRI-based neuroimaging

Antibodies

Antibodies used	WB antibodies: anti-HA (Sigma-Aldrich, H3663, monoclonal Antibody), anti-ZSCAN4 (Millipore, AB3430,Polyclonal Antibody), anti-ubi (CST, Ubiquitin (P4D1) MousemAb#3936, monoclonal Antibody) and anti-GAPDH (Abclonal, AC033, AMC0062). These antibodies were all used at 1:2000 dilution. The second antibody conjugated with HRP (Genescrip, A00098, Polyclonal Antibody) and used as 1:5000 dilution.<br>CHIP antibody: anti-HA (Santa, sc-7392,monoclonal Antibody). 3ug antibody was used for one pulldown.
Validation	The following antibodies are from commercial sources and have been validated in our previous studies. The sources of the antibodies are<br>anti-HA (https://www.sigmaaldrich.cn/CN/zh/product/sigma/h3663)<br>anti-ZSCAN4 (https://www.merckmillipore.com/CN/zh/product/Anti-Zscan4-Antibody,MM_NF-AB4340)<br>anti-GAPDH (https://abclonal.com.cn/catalog/AC033)<br>anti-HA (https://datasheets.scbt.com/sc-7392.pdf)<br>anti-ubi (https://www.cellsignal.cn/products/primary-antibodies/ubiquitin-p4d1-mouse-mab/3936?site-search-type=Products&N=4294956287&Ntt=3936&fromPage=plp&_requestid=1478915)<br>The ZFP352 antibody was generated in house in this study and validated by western blotting upon exogenous ZFP352 over-expression, siRNA knocking down and immuno-staining of ZFP352 in mouse embryos.

Eukaryotic cell lines

Policy information about cell lines and Sex and Gender in Research

Cell line source(s)	HEK293T cells are from Pricella, CL-0005;<br>E14 cells (Cell Search System, E14tg2a) are a gift from Shen Li lab.
Authentication	All the cell lines were routinely authenticated by morphology check using microscope.
Mycoplasma contamination	The cell lines are routinely tested, and they are negative for Mycoplasma contamination.
Commonly misidentified lines<br>(See ICLAC register)	No commonly misidentified cell lines were used in this study.

Animals and other research organisms

Policy information about studies involving animals; ARRIVE guidelines recommended for reporting animal research, and Sex and Gender in Research

Laboratory animals	The 8-10 weeks old female ICR mice were purchased from Shanghai SLAC Laboratory Animal Co., Ltd. All the mice were housed under the SPF environment with a 12 hour light-dark cycle, and had a temperature of 22-24C with 50–60% humidity.
Wild animals	No wild animals were used in the study.
Reporting on sex	No Reporting on sex were used in the study.
Field-collected samples	No field-collected samples were used in the study.

Ethics oversight	Zhejiang University (China) provided the guidance for the animal research protocol with the ethical approval number as ZJU20230182.

Note that full information on the approval of the study protocol must also be provided in the manuscript.

ChIP-seq

Data deposition

☒ Confirm that both raw and final processed data have been deposited in a public database such as GEO.

☒ Confirm that you have deposited or provided access to graph files (e.g. BED files) for the called peaks.

Data access links<br>*May remain private before publication.*	The ChIP-seq data are available at website: https://www.ncbi.nlm.nih.gov/geo/ with the accession and token as below:<br>GSE202158:ohwxoiwwppqztqh<br>GSE222634:ovidokusfbmljmp
Files in database submission	GSM6102001: Zfp352-OE, input;<br>GSM6102002: Zfp352-OE, pull-down with ZFP352 antibody;<br>GSM6928074: Zfp352OE,input;<br>GSM6928075: Zfp352OE,pull-down with ZFP352 antibody;<br>GSM6928076: Zfp352+DuxOE,input;<br>GSM6928077: Zfp352+DuxOE,ZFP352,pull-down with ZFP352 antibody
Genome browser session<br>(e.g. UCSC)	N.A.

Methodology

Replicates	One
Sequencing depth	GSM6102001: total number of reads: 161.51 million; uniquely mapped reads: 158.2 million; length of reads: 100; single-end.<br>GSM6102002:total number of reads: 170.48million; uniquely mapped reads: 168.47 million; length of reads: 100; single-end.<br>GSM6928074:total number of reads: 132 million; uniquely mapped reads: 119.1 million; length of reads: 150; paired-end.<br>GSM6928075:total number of reads: 116.2 million; uniquely mapped reads: 114.3 million; length of reads: 150; paired-end.<br>GSM6928076:total number of reads: 140.35 million; uniquely mapped reads: 137.93 million; length of reads: 150; paired-end.<br>GSM6928077:total number of reads: 103.07 million; uniquely mapped reads: 100.66 million; length of reads: 150; paired-end.
Antibodies	anti-HA (Santa,sc-7392)
Peak calling parameters	callpeak -t ZFP_IP_R1.mLb.clN.sorted.bam -c INPUT_R1.mLb.clN.sorted.bam -f BAM -g 1.87e9 -n ZFP_IP_R1 --keep-dup all
Data quality	GSE202158:FDR<0.05, Fold enrichment >5: 10401 peaks<br>GSE222634:FDR<0.05, Fold enrichment >5: Zfp352OE:ZFP-IP:4071 peaks;Zfp352+DuxOE-ZFPIP:5917 peaks
Software	bioinformatics pipelines “nf-core/chipseq” (v1.1.0)

Flow Cytometry

Plots

Confirm that:

☒ The axis labels state the marker and fluorochrome used (e.g. CD4-FITC).

☒ The axis scales are clearly visible. Include numbers along axes only for bottom left plot of group (a 'group' is an analysis of identical markers).

☒ All plots are contour plots with outliers or pseudocolor plots.

☒ A numerical value for number of cells or percentage (with statistics) is provided.

Methodology

Sample preparation	mESCs were collected and fixed 30min with 4% PFA after wash twice with PBS.
Instrument	BD Fortessa
Software	FlowJo_V10
Cell population abundance	For analysis of the MERVL +ve cells, each time at least 10-20K cells were measured to determine the percentage of MERVL +ve population.

Gating strategy

The GFP+ and mCherry+ gates were drawn using the untransfected or uninduced cells as controls.

☒ Tick this box to confirm that a figure exemplifying the gating strategy is provided in the Supplementary Information.
